# Mutation Enrichment and Transcriptomic Activation Signatures of 419 Molecular Pathways in Cancer

**DOI:** 10.3390/cancers12020271

**Published:** 2020-01-22

**Authors:** Marianna A. Zolotovskaia, Victor S. Tkachev, Alexander P. Seryakov, Denis V. Kuzmin, Dmitry E. Kamashev, Maxim I. Sorokin, Sergey A. Roumiantsev, Anton A. Buzdin

**Affiliations:** 1 Oncobox Ltd., Skolkovo Innovation Center, 121205 Moscow, Russia; buzdin@oncobox.com; 2 Department of Oncology, Hematology and Radiotherapy of Pediatric Faculty, Pirogov Russian National Research Medical University, 117997 Moscow, Russia; s_roumiantsev@mail.ru; 3 Moscow Institute of Physics and Technology, Dolgoprudny, 141701 Moscow Region, Russia; kuzmin.dv@mipt.ru; 4 Omicsway Corp., Office 6040, Walnut, CA 91789, USA; tkachev@oncobox.com (V.S.T.); sorokin@oncobox.com (M.I.S.); 5 Medical Holding SM-Clinic, 105120 Moscow, Russia; alseryakov@yandex.ru; 6 The Laboratory of Clinical Bioinformatics, I.M. Sechenov First Moscow State Medical University, 119991 Moscow, Russia; dkamashev@gmail.com; 7 Shemyakin-Ovchinnikov Institute of Bioorganic Chemistry, 117997 Moscow, Russia

**Keywords:** cancer, DNA mutation, intracellular molecular pathways, carcinogenesis, transcriptome, molecular pathway activation

## Abstract

Carcinogenesis is linked with massive changes in regulation of gene networks. We used high throughput mutation and gene expression data to interrogate involvement of 278 signaling, 72 metabolic, 48 DNA repair and 47 cytoskeleton molecular pathways in cancer. Totally, we analyzed 4910 primary tumor samples with individual cancer RNA sequencing and whole exome sequencing profiles including ~1.3 million DNA mutations and representing thirteen cancer types. Gene expression in cancers was compared with the corresponding 655 normal tissue profiles. For the first time, we calculated mutation enrichment values and activation levels for these pathways. We found that pathway activation profiles were largely congruent among the different cancer types. However, we observed no correlation between mutation enrichment and expression changes both at the gene and at the pathway levels. Overall, positive median cancer-specific activation levels were seen in the DNA repair, versus similar slightly negative values in the other types of pathways. The DNA repair pathways also demonstrated the highest values of mutation enrichment. However, the signaling and cytoskeleton pathways had the biggest proportions of representatives among the outstandingly frequently mutated genes thus suggesting their initiator roles in carcinogenesis and the auxiliary/supporting roles for the other groups of molecular pathways.

## 1. Introduction

Cancer has complex pathogenesis [1] and molecular mechanisms underlying its development and progression still remain underexplored [2]. According to the somatic mutation theory, the key point in cancer transformation is DNA damage, e.g., due to aberrantly regulated or nonfunctional DNA repair pathways [3]. Cancer cells accumulate mutations several times faster than the normal cells [4]. It contributes to deregulation of molecular pathways including those responsible for apoptosis, cell growth, metabolism, motility and immunosuppression [5,6]. In turn, this leads to epigenetic changes that promote further malignization, e.g., through the repression of tumor suppressor genes [7].

However, the analysis of individual genes *per se* is not enough for understanding mechanisms of carcinogenesis because each gene product only serves as a component of complex biological processes determining cell fate. One of the key systemic genetic approaches in cancer is data analysis at the level of molecular pathways [8,9,10,11]. It was found previously that changes at the molecular pathway levels characterize cancers better than individual gene mutations and expression levels due to greater stability and reliability of the additive biomarkers [12,13,14].

Several databases of molecular pathways were published, frequently specifically devoted to the pathways with specific functions, such as MetaCyc and SynSysNet [15,16]. On the other hand, a more universal database like Kyoto Encyclopedia of Genes and Genomes (KEGG) classifies the pathways by relation to “metabolism, processing of genetic information, processing of environmental information, cellular processes, organizational systems, diseases and drug development” [17]. Many attempts to investigate cancer interactomes on the basis of molecular pathways have been made, showing mosaic picture of tumor-associated lesions [18,19,20,21,22]. In this study, we aimed to perform the most comprehensive analysis of the molecular pathway’s mutation and activation features in cancer. To this end, we used new instruments to algorithmically assess pathway activation levels [23] and pathway mutation instability rates [24] by analyzing high throughput gene expression and whole exome sequencing data. To our knowledge, these metrics were never systemically investigated before for simultaneous large-scale characterization of molecular pathways in cancer. Using descriptions provided by the pathway database administrators and available literature, we classified 419 available molecular pathways into four functional groups of *signaling*, *metabolic*, *cytoskeleton* and *DNA repair* processes (including 278, 72, 47 and 48 pathways, respectively).

The *signaling pathways* transmit different types of signals into the cell, e.g., signals from external cell surface receptors. They control all major biological processes in the cell such as proliferation, cell growth, migration, differentiation and death. Impaired molecular signaling can lead to acquisition of cancer phenotype by the cell and further disease progression [25,26,27,28].

*Metabolic pathways* are responsible for the whole repertoire of biochemical reactions in organism. In the living cell they are dynamically controlled by the external signals, concentrations of biomolecules and differentiation programs [29]. In cancer, the regulation of metabolism is strongly biased due to increased consumption of energy required for forced proliferation [30,31]. For example, Warburg effect of replacing oxidative phosphorylation as the major provider of ATP by glycolysis in cancer cells is well known since 1920s [32]. On the other hand, cancer cells frequently de-differentiate and abrogate their specialized molecular functions, thus losing complexity of metabolic patterns [33].

In turn, the *cytoskeleton* and *DNA repair* pathways may be considered as the specific sort of signaling pathways because many of them if not all are directly controlled by external or internal stimuli [34,35]. Imbalanced *cytoskeleton pathways* can lead to defects in mitosis and cellular morphogenesis, altered intercellular contacts and cell motility. This can promote cancer progression, invasion and metastasis [36]. 

Finally, mutations in *DNA repair* pathways are most likely the key mechanisms in the emergence and development of malignant tumors. Mutations in genes responsible for genome integrity frequently point on individuals with a predisposition to cancer, but also may serve as biomarkers of response on specific anticancer therapies [37]. These mutations also induce multiplicative tumorigenic effects because they initiate accelerated molecular evolution of cancer cells, that can both repress tumor suppressor genes and upregulate oncogenes, while escaping restriction by the immune system [38,39]. Moreover, a link was shown between functional changes in DNA repair genes and switching of cancer cell metabolic programs [40,41].

In this study we for the first time scrutinized mutation frequencies and expression profiles of genes included in 419 molecular pathways belonging to the above four groups with different biological functions. Totally, we analyzed 4910 matched individual cancer gene expression and whole exome sequencing profiles collectively covering 1,252,669 mutations from the TCGA project database [42] in thirteen cancer types. We identified no correlation between mutation enrichment and expression changes both at the individual gene and molecular pathway levels. However, we found that pathway activation profiles were largely congruent among the different cancer types. Overall, positive median cancer-specific activation levels were seen in the DNA repair, versus similar slightly negative values in the other types of pathways. The DNA repair pathways also demonstrated the highest values of mutation enrichment. However, the signaling and cytoskeleton pathways had the biggest proportions of representatives among the outstandingly frequently mutated genes thus suggesting their initiator roles in carcinogenesis and the auxiliary/supporting roles for the other groups of molecular pathways. 

For all the biosamples, we present a database of mutation and expression data at the individual gene and at the molecular pathway levels. At the gene level, normalized mutation rate and case-to-normal ratio data are given. Conversely, at the pathway level we present algorithmically calculated pathway mutation instability rates [24] and pathway activation levels [23], respectively.

## 2. Results

### 2.1. Cancer Mutation Frequencies of Molecular Pathway Genes

We extracted molecular pathway gene contents and architecture from the publicly available databases [17,43,44,45,46,47] and refined by expert manual curation. Were classified the pathways extracted into four major functional groups: signaling (278 members), metabolic (72 members), and DNA repair (48 members), cytoskeleton pathways (47 members), listed in Supplementary Table S1. Twenty-six pathways were simultaneously classified as the signaling and DNA repair pathways, and our further analyses we used only the 419 non-overlapping molecular pathways. 

**Figure 1:**
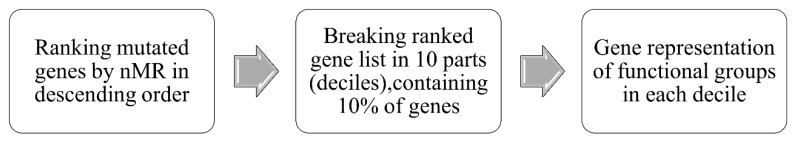
Pipeline for bioinformatic analysis of gene mutation frequencies corresponding to functional groups of molecular pathways.

We then measured mutation rates of genes belonging to these functional groups of pathways (Supplementary Table S2). To this end, a complete list of non-synonymous somatic mutations mapped in 19,608 genes in 4910 tumor samples was built and processed according to the analytic pipeline outlined in Figure 1. 

The tumor samples analyzed represented thirteen tumor types and were originally profiled within the The Cancer Genome Atlas (TCGA) project [42] while the respective whole exome mutation profiles were taken from the Catalogue Of Somatic Mutations In Cancer (COSMIC) database [48]. Normalized mutation rates (nMR) of all genes investigated are listed in Supplementary Table S3. 

**Figure 2:**
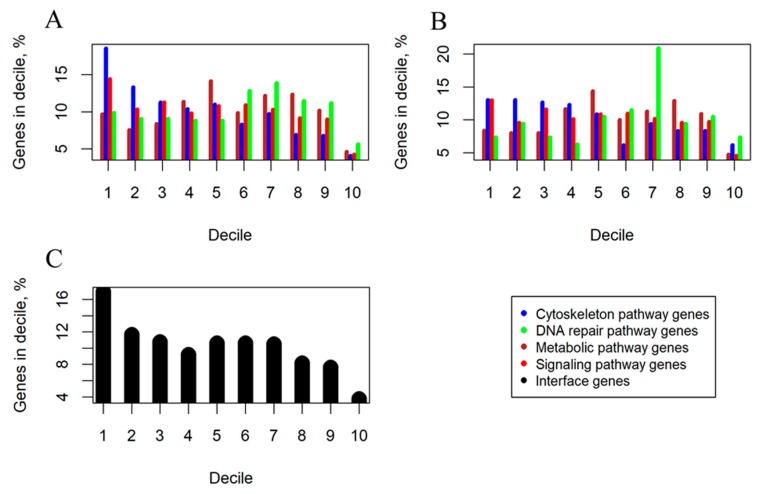
Decile analysis of cancer-associated mutations (deciles ordered from high gene mutation rate on the left to low on the right). (**A**) Percentage shares of genes belonging to signaling, metabolic, cytoskeleton, DNA repair pathway functional groups. (**B**) Percentage shares of pathway group-specific, non-overlapping genes. (**C**) Percentage shares of genes present in two or more functional groups of molecular pathways (termed interface genes).

The nMR values were ranked in the descending order and then divided into 10 parts (deciles), each representing 10% of genes. In each decile, we measured proportions of genes belonging to the four functional groups of pathways under investigation (Figure 2). We observed a trend that the *signaling* and *cytoskeleton* pathways had highest representations in the first decile which was decreasing further to the minimal values in the last decile, thus evidencing high proportion of especially frequently mutated genes in these groups (Figure 2). In contrast, the *metabolic* and *DNA repair* pathways showed a different trend, where maximum representations were reached in the central deciles, with relatively modest representations in the first and in the last deciles (Figure 2).

**Figure 3:**
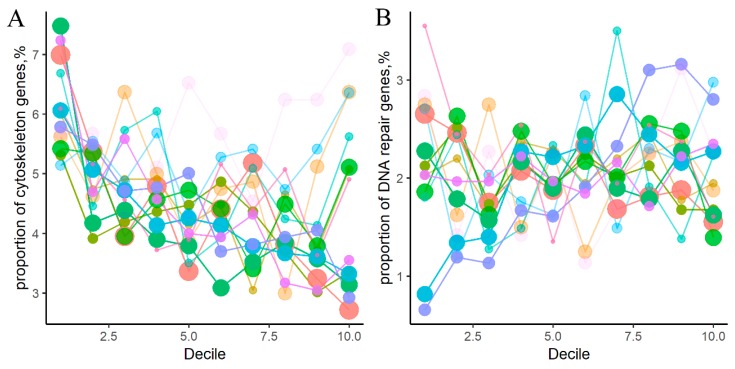

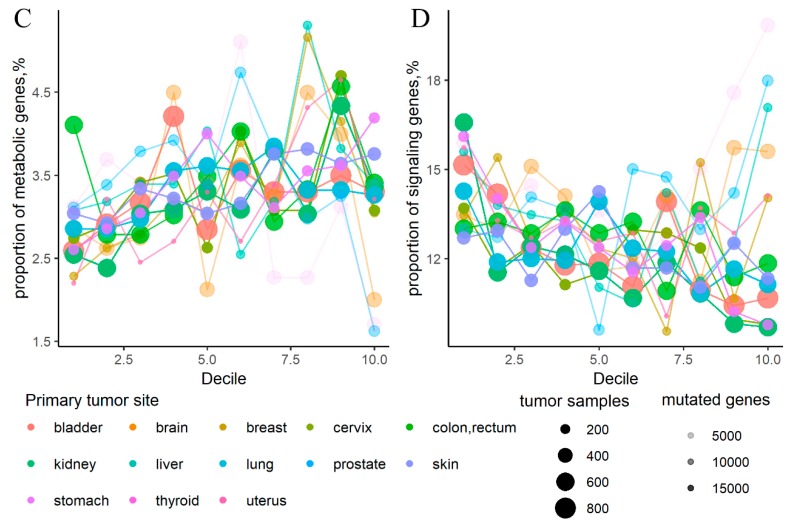
Percentage share in different cancer types of genes belonging to four groups of molecular pathways: (**A**) cytoskeleton, (**B**) DNA repair, (**C**) metabolic, (**D**) signaling pathways. Color represents cancer type. Color density is proportionate to number of mutant genes per group per decile. Point width reflects number of samples in cancer type.

We then re-analyzed these representations and confirmed the above two trends separately for the thirteen cancer types (Figure 3; Supplementary Table S4). These trends were more pronounced for the cancer types represented by bigger number of samples, most probably, due to greater statistical power (Figure 3).

**Figure 4:**
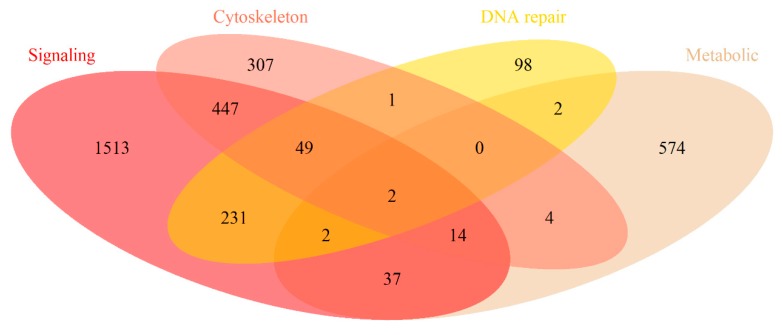
Intersection of gene composition for different functional groups of molecular pathways.

To characterize in brief gene compositions of the molecular pathways, we intersected them for the different groups of pathways (Figure 4). Jaccard (JC) and Szymkiewicz-Simpson (SS) [49] similarity coefficients between the pathway groups are shown on Table 1. 

In total, all the 419 pathways contained 3281 unique genes, of them 809 were the interface genes, i.e., simultaneously members of two or more groups of pathways. *Signaling* pathways included 2295 genes, of them 1513 were specific for this group and the rest were the interface genes. *Metabolic* pathways had totally 635 genes and 574 specific genes; *cytoskeleton* pathways—824 and 307 genes, respectively; *DNA repair* pathways—385 and 98 genes, respectively. The highest intersection level was seen for the *signaling* and *cytoskeleton* pathway genes (JC = 0.196, SS = 0.621) and for the *signaling* and *DNA repair* genes (JC = 0.119, SS = 0.738).

To avoid any possible bias linked with the interface genes, we then repeated the decile distribution analysis for the fraction of pathway-specific, non-overlapping genes. The decile-specific gene distribution trends previously seen for the *signaling*/*cytoskeleton* vs *metabolic*/*DNA repair* groups of pathways were also clearly detectable for the pathway type-specific genes (Figure 2B). Moreover, the fraction of interface genes that was mostly formed by genes from the intersection of *signaling* and *cytoskeleton* groups of pathways also showed the same trend as for the *signaling*/*cytoskeleton* groups (Figure 2C). Interestingly, the interface genes had overall higher mutation rates than the pathway type-specific genes (Figure 2B,C), which can relate to their relatively more important functions in tumorigenesis, e.g., because of their simultaneous participation in different types of molecular processes.

Every tumor sample investigated had mutated genes. Among them, 78% of samples had at least *one* mutated gene from cytoskeleton, 53%—from DNA repair, 58%—from metabolic, and 94%—from signaling pathways (Table 2). Moreover, 65% of tumor samples had at least *two* mutated genes from cytoskeleton, 35%—from DNA repair, 41%—from metabolic, and 89%—from signaling pathways, and so on up to 20 mutated genes, further showing prevalence of signaling and cytoskeleton pathways (Table 2). 

### 2.2. Mutation Enrichment and Pathway Activation Levels

The above 4910 tumor samples were selected for the pathway analysis based on simultaneous availability of both RNA sequencing and whole exome mutation profiles. In addition, from the TCGA data repository we extracted the gene expression profiles for 655 tumor-matched adjacent normal tissue samples. These normal samples represented the same localizations/tissue types as the corresponding primary tumor samples (Supplementary Table S5). 

We then used tumor and normal RNA sequencing profiles to analyze differential gene expression and to assess activation levels of molecular pathways. For every comparison, the tumor profiles were normalized on the corresponding normal tissues, e.g., breast cancer profiles were normalized on the normal breast samples, etc. *Pathway activation level* (*PAL*) values were calculated to characterize molecular pathway activation using differential gene expression data [23,50,51], Supplementary Table S6. *PAL* scores can take positive and negative values and are congruent with the expected differential activation of a pathway [23].

The mutation enrichment of the same molecular pathways in 4910 tumors was assessed using *Pathway instability* (*PI*) metric that characterizes average mutation load per gene in a pathway [14], Supplementary Table S7. *PI* score can take only positive values and is congruent with the pathway mutation burden.

**Figure 5:**
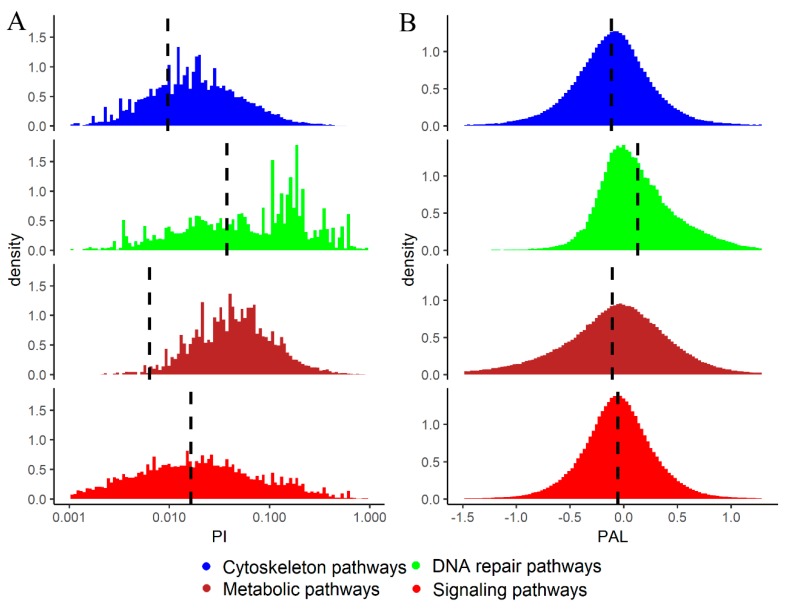
(**A**) Distributions of PI > 0 scores for cytoskeleton, metabolic, DNA repair and signaling molecular pathways in 4910 cancer samples. PI axis is given in logarithmic scale. Vertical dashed lines show mean PI values calculated for all cases including PI = 0. (**B**) Distributions of PAL scores for cytoskeleton, metabolic, DNA repair and signaling molecular pathways in the same cancer samples. Vertical dashed lines show mean PAL values calculated for the respective group of pathways for all tumor samples.

In the tumor samples investigated, 32.3% of *signaling* pathways, 30.4% of *cytoskeleton* pathways, 29.2% of *DNA repair* pathways, and 9.6% of *metabolic* pathways had mutations in their genes and, therefore, had PI values greater than zero. Interestingly, the distribution of PI scores demonstrated that overall the *DNA repair* pathways had the highest relative mutation rates per gene, the *signaling* pathways had intermediate scores and the *metabolic* and *cytoskeleton* pathways had the lowest relative mutation levels (Figure 5A).

**Figure 6:**
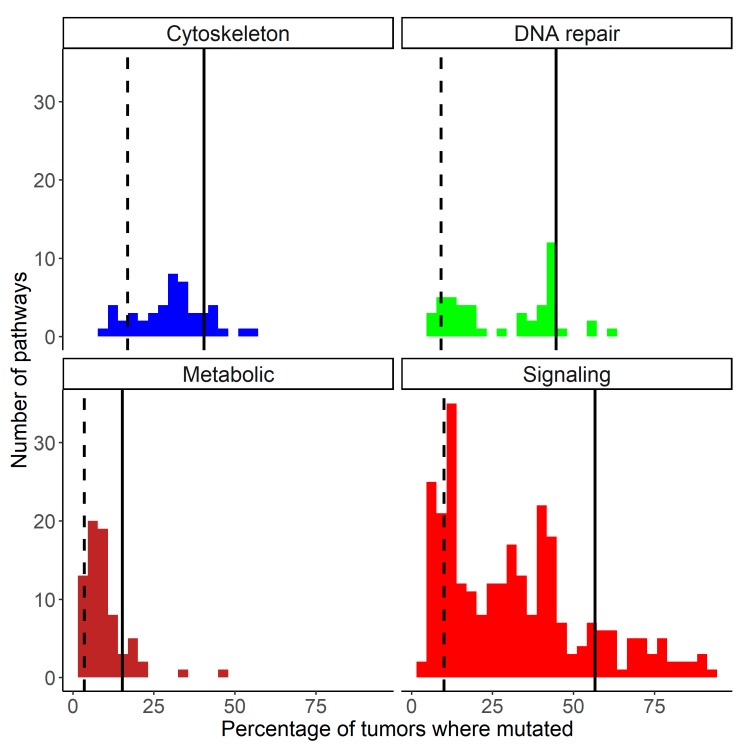
Distributions of molecular pathways according to percentage of tumor samples having mutations within them, for cytoskeleton, DNA repair, metabolic, signaling pathways. Vertical solid lines delineate 15% most frequently mutated pathways (**on the right**), vertical dashed lines delineate 15% least mutated pathways (**on the left**).

**Figure 7:**
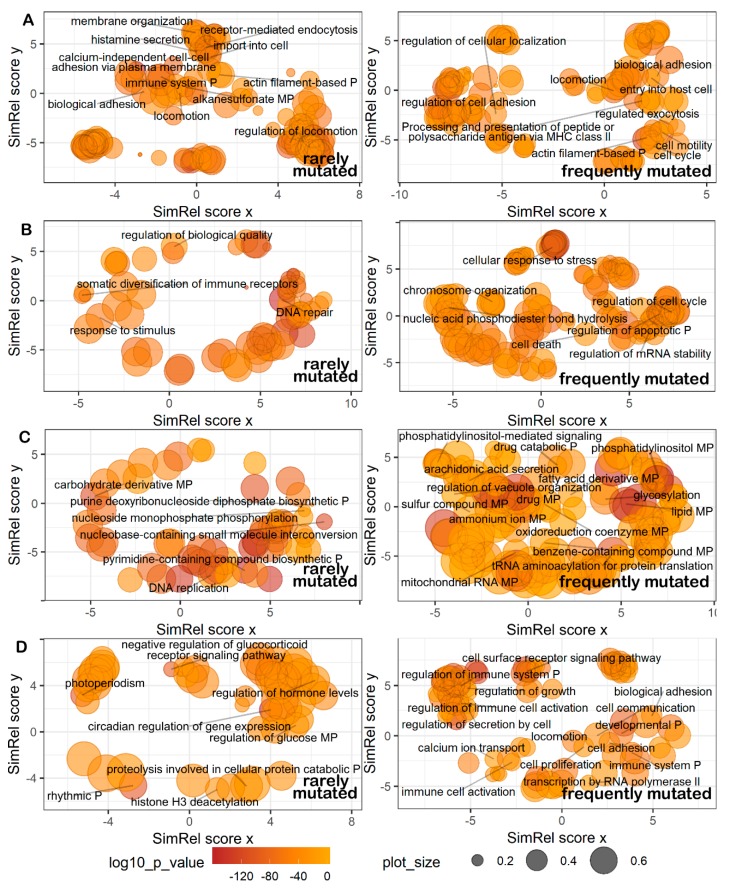
Representation of Gene Ontology annotations for genes from 15% least (**left**) and 15% most frequently (**right**) mutated (**A**) cytoskeleton, (**B**) DNA repair, (**C**) metabolic, (**D**) signaling pathways. Bubble size indicates the frequency of the gene ontology term in the underlying GOA database [54] (bubbles corresponding to more general terms are shown larger). “P” stands for process, “MP”—for metabolic process. Too general and non-informative gene ontology terms are not defined.

We then investigated in more details what biological processes were connected with the genes from the most and least mutated pathways. To this end, for every type of pathways we took top and bottom 15% pathways (Figure 6; Supplementary Table S8). To identify relevant Gene Ontology terms, the gene lists from these top and bottom pathways were analyzed using GOrilla software [52]. The obtained terms were then plotted with REVIGO software [53], Figure 7. The least mutated pathways (Figure 7A–D, left panels) dealt (*cytoskeleton*, Figure 7A) with membrane organization, receptor-mediated endocytosis, actin filament binding and locomotion, calcium-independent cell-cell adhesion via plasma membrane; (*DNA repair*, Figure 7B) with regulation of DNA quality and associated DNA repair, and with rearrangement of immunoglobulin loci; (*metabolic*, Figure 7C) with biosynthesis of nucleotides and DNA replication; (*signaling*, Figure 7D) with negative regulation of glucocorticoid receptor pathway, regulation of hormone levels, circadian rhythms, H3 histone deacetylation and protein catabolism. 

In turn, the most strongly mutated pathways (Figure 7A–D, right panels) were responsible for (*cytoskeleton*, Figure 7A) cellular adhesion and cell surface receptor binding, exocytosis, cell motility, cell communication and locomotion, cell cycle progression, processing and presentation of antigens via the major histocompatibility complex (MHC )class II; (*DNA repair*, Figure 7B) cellular response to stress, chromosome organization, hydrolysis of phosphodiester bonds; (*metabolic*, Figure 7C) drug response and catabolism, arachidonic acid secretion, fatty acid derivative pathways, glycosylation, sulfur and benzene-containing compound pathways, production of mitochondrial RNA, and tRNA aminoacetylation for protein translation; (*signaling*, Figure 7D) regulation through phosphatidyl inositol, cell surface receptor pathways, growth and proliferation, regulation of calcium transport, transcription by RNA polymerase II, and regulation of immune cell activation.

At the level of pathway activation (PAL scores), most of the pathway types were mostly downregulated in cancer, except for the *DNA repair* pathways that were mostly upregulated (Figure 5B). The general downregulation observed was maximal for the *cytoskeleton* pathways (Figure 5B). Overall, averaged PAL scores showed cancer-specific activation in approximately 60.3% of *DNA repair*, 44.7% of *metabolic*, 42.8% of *signaling* and 37.5% of *cytoskeleton* pathways. 

**Figure 8:**
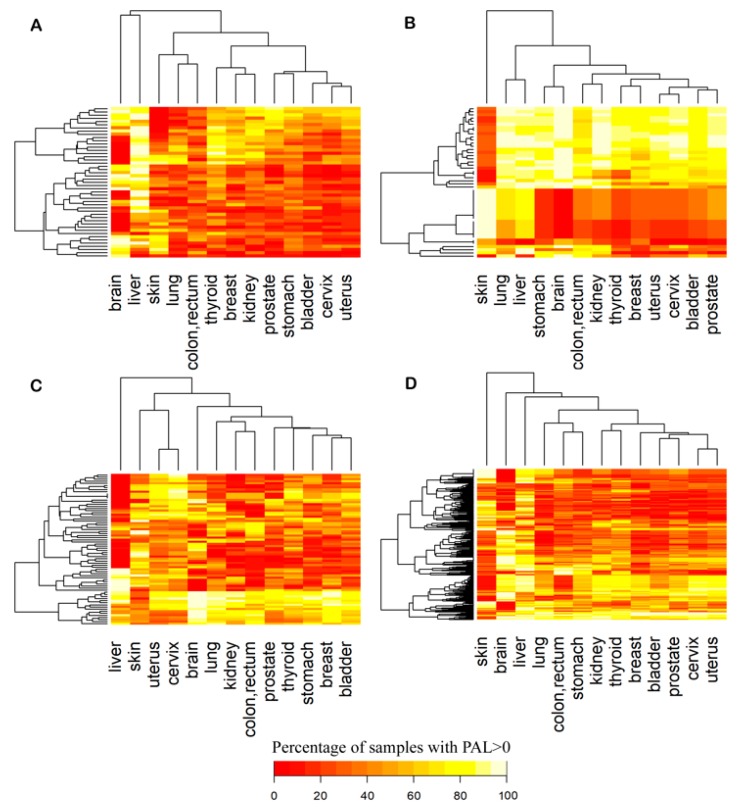
Percentage shares of cancer samples with PAL > 0 in thirteen cancer types for: (**A**) cytoskeleton, (**B**) DNA repair, (**C**) metabolic, (**D**) signaling pathways under investigation. For every pathway, color scale corresponds to percentage of samples where it is upregulated (PAL > 0).

We then investigated in more detail up- and downregulated pathways in each group. Because the same pathway can be differently regulated in different samples, we tried to focus on cancer type-specific changes. To this end, for every pathway in every cancer type, we calculated percentage of samples in which the pathway was upregulated, as reflected by PAL > 0 (Figure 8). Most of DNA repair pathways were activated in the majority of samples (Figure 8B), whereas cytoskeleton, metabolic and signaling pathways showed mosaic picture (Figure 8A,C,D). We also found that most of the pathways showed uniform, cancer type-independent activation pattern in tumor samples (Figure 8). 

**Figure 9:**
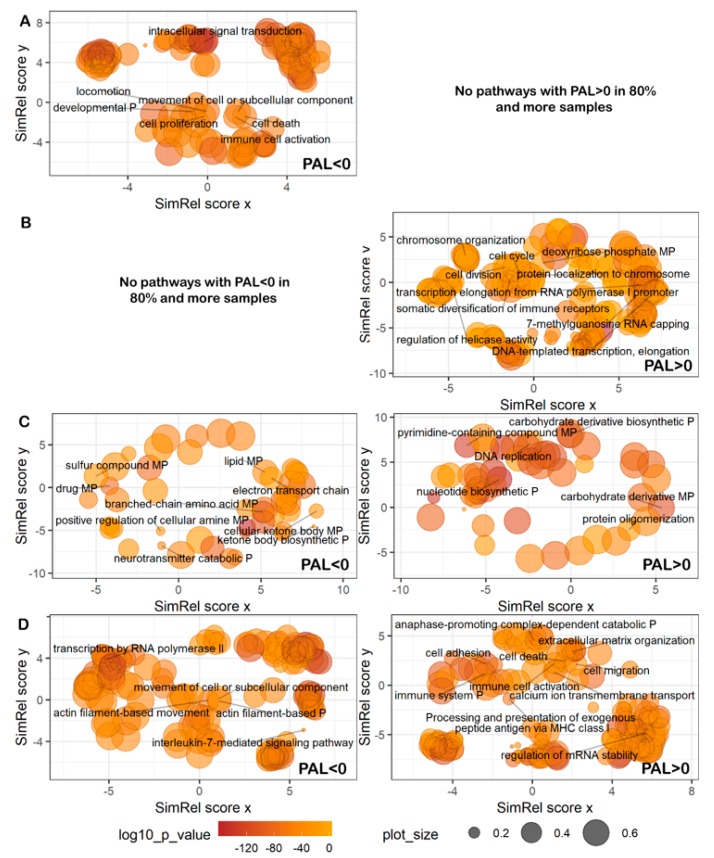
Representation of Gene Ontology annotations for genes from most strongly downregulated (PAL < 0; left) and upregulated (PAL > 0; right) (**A**) cytoskeleton, (**B**) DNA repair, (**C**) metabolic, (**D**) signaling pathways. Bubble size indicates the frequency of the Gene Ontology term in the underlying GOA database [54] (bubbles corresponding to more general terms are shown larger). “P” stands for process, “MP”—for metabolic process. Too general and non-informative gene ontology terms are not defined. No cytoskeleton pathways with PAL < 0 and DNA repair pathways with PAL > 0 could be identified in 80% and more samples.

We then performed Gene Ontology analyses of up/downregulated pathways. To this end, we took fractions of top pathways with PAL > 0 in more than 80% of cancer samples under investigation, and of bottom pathways with PAL < 0 in more than 80% of the samples. In this way we selected totally 8 up/14 downregulated signaling pathways, 4/2 metabolic pathways, 15/0 DNA repair pathways, and 0/2 cytoskeleton pathways, accordingly. As before, the genes from these pathways were subjected for gene ontology analysis using GOrilla software [52] the results of which were visualized using REVIGO [53] viewer (Figure 9; Supplementary Table S9). The most strongly cancer-downregulated pathways related to (*cytoskeleton*, Figure 9A) movement of cell or subcellular components and locomotion, regulation of development and cell proliferation; (*metabolic*, Figure 9C) cellular ketone body metabolism, neurotransmitter catabolic processes, sulfur pathways, drug response pathways; (*signaling*, Figure 9D) regulation of transcription by RNA polymerase II, interleukin-7-mediated pathway. We found no strongly cancer-downregulated *DNA repair* pathways.

Among the most strongly cancer-upregulated pathways (Figure 9A–D, right panels), there were no *cytoskeleton* pathways, whereas the others dealt with (*DNA repair*, Figure 9B) response to DNA templated transcription and elongation, 7-methylguanosine RNA capping, protein localization on chromosomes and chromosomal organization, rearrangement of immune receptor loci; (*metabolic*, Figure 9C) oligosaccharide biosynthesis and protein oligomerization, nucleotide biosynthesis and DNA replication; (*signaling*, Figure 9D) cell migration and adhesion, regulation of cell death, calcium ion transmembrane transport, extracellular matrix organization, regulation of mRNA stability, processing and presentation of exogenous peptide antigen via MHC class I, and activation of immune cells.

Remarkably, we found no connection between mutation burden (PI scores) and the respective activation of the specific molecular processes (PAL scores). For example, the processes of transcription by RNA polymerase II, chromosomal organization, processing and presentation of antigens via MHC class II and activation of immune cells were featured for the genes from *strongly mutated* and at the same time *upregulated* molecular pathways. The drug response, sulfur pathway categories included genes from *strongly mutated*, *downregulated* pathways. The rearrangement of immunoglobulin loci, biosynthesis of nucleotides, DNA replication, and circadian rhythmic processes involved genes of *poorly mutated*, *upregulated* pathways. 

**Figure 10:**
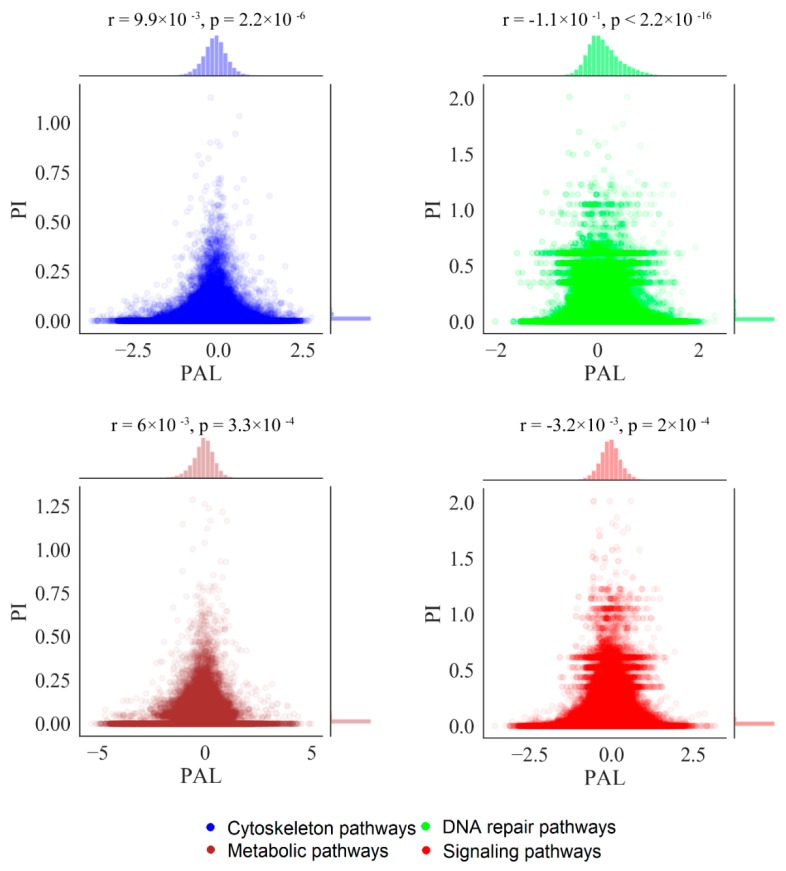
Correlation of PI and PAL scores for different types of molecular pathways in 4910 tumor samples.

For all types of the pathways, we found no correlation between mutation burden and expression changes at the level of individual genes, i.e., case-to-normal ratio (CNR) and normalized mutation rate (nMR) values (Supplementary Figure S1). We also found no correlation at the pathway level—between the PAL and PI values, for all four pathway types (Figure 10). 

**Figure 11:**
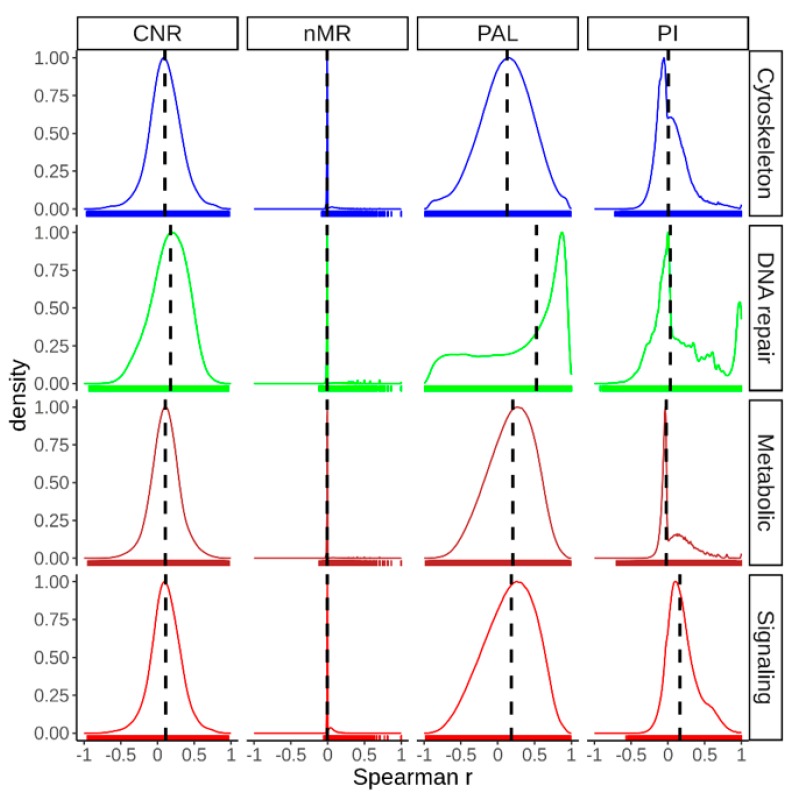
Distribution of pairwise Spearman correlation coefficients between all possible combinations of tumor samples under investigation for mutation and expression data at both gene wise (nMR, CNR) and pathway (PAL, PI) level in cytoskeleton, DNA repair, metabolic and signaling pathway groups. Vertical dashed lines indicate the medians of the distributions.

We then assessed correlations between all tumor samples for mutation burden at gene (nMR) and pathway (PI) levels, and also for expression changes at gene (CNR) and pathway (PAL) levels (Figure 11). To this end for every pair of tumor samples we calculated pairwise Spearman correlation coefficient, separately in all four pathway groups (Figure 11). For mutation signatures, the samples were highly heterogeneous with near-zero medians of Spearman correlation at the gene (nMR) and the pathway (PI) levels, except for slightly higher PI of signaling pathways (Spearman correlation 0.16). In contrast, for gene expression, the medians of Spearman correlation coefficients varied from 0.1 till 0.18 at the gene level (CNR), and much higher at the pathway level (PAL): from 0.12 till 0.52. These results evidence significantly greater inter-tumoral similarity at the level of pathway activation compared to the individual gene expression level and, especially, in comparison with both types of mutational data (Figure 11). These trends were also confirmed in thirteen analyses performed for all separate cancer types (Supplementary File S1). Interestingly, in most of the analyses, intertumoral activation profiles of the DNA repair pathways showed significantly higher congruences than it was observed for the other pathway types (Figure 11, Supplementary File S1).

Taken together, these data suggest that for both mutation and gene expression data, the intertumoral similarities are much higher at the pathway level (Figure 11, Supplementary File S1). On the other hand, DNA repair pathways demonstrate the most congruent activation patterns among the tumors. 

## 3. Discussion

Juxtaposition of clinical and molecular tumor phenotypes provides a basis for further improvement of cancer treatments. High-throughput cancer gene expression and mutation data can help identifying new oncogenes and driver mutations [55]. In turn, further analysis of cancer data at the level of molecular pathways helps understanding pathological molecular changes in a quantitative way [50,56,57,58]. 

Nowadays, many molecular pathways were reported and collected in specific databases. Despite the existence of universal methods to pathway annotation like PathwayCommons, these databases were mostly generated separately and utilize different approaches to pathways nomenclature, and the same or very similar pathways can be included in different databases under different names [59]. Functional classification of the pathways also depends on a database, e.g., HumanCyc repository contains only metabolic pathways [45]. The structure and composition of the different pathway databases are continuously being revised in search for uniformity and comprehensiveness [59,60]. 

However, most studies of cancer biology focus on only single pathways or small groups of pathways [61,62]. For example, in 2018, a detailed study of the mechanisms and patterns of somatic alterations in 10 signaling pathways in 33 cancer types revealed patterns of co-occurrence and mutual exclusivity, driver changes, single and multiple potentially targeted mutations [22]. However, high-throughput simultaneous comparison of mutation and activation features of different types of molecular pathways was missing [63,64].

In this study, we performed more thorough analysis in terms of number of molecular pathways investigated. We explored 419 molecular pathways using new instruments to algorithmically assess pathway activation levels [23] and pathway mutation instability scores [24] by analyzing high throughput gene expression and whole exome sequencing data. To our knowledge, these metrics were never systemically investigated before for simultaneous large-scale characterization of molecular pathways in cancers. This enabled us to perform the comprehensive comparative characterization of four major functional groups of pathways in thirteen human cancer types. For the first time, for thirteen cancer primary localizations we investigated general mutation and activation features specifically for each type of the *signaling*, *cytoskeleton*, *metabolic* and *DNA repair* pathways. 

**Figure 12:**
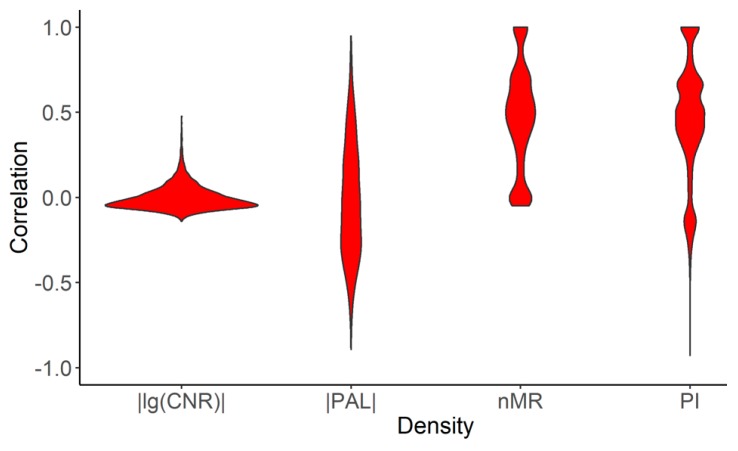
Distribution of correlation coefficients calculated for 4382 tumor samples for ten signaling pathways from [22] and enclosed individual genes between: (*i*) binary alteration score (BAS) at the gene level with absolute values of lg(CNR) and normalized mutation rate (nMR); (*ii*) BAS at the pathway level and absolute values of pathway activation level (PAL) and pathway instability (PI).

We then compared our results with the previous study “Oncogenic Signaling Pathways in The Cancer Genome Atlas” on genetic alterations scoring in ten signaling pathways using TCGA data [22]. The pathways were ranked on the basis of specific alteration score, where genes in each individual tumor were defined as either altered or not altered. Altered genes had at least one of the following: copy-number alterations, mutations, fusions or specific features of epigenetic silencing. Consequently, altered pathway was defined as pathway having at least one altered gene. Alterations (binary alteration score, BAS) were marked as binary values. Therefore, binary alteration scores were calculated both at the gene and the pathway levels. Totally 9125 tumor samples of 38 tumor subtypes were investigated, among them 4382 were also investigated in our study. To compare the results obtained, for those overlapping 4382 tumor samples, we calculated our functional metrics (nMR, CNR, PAL, PI) for the same ten pathways that were investigated in the previous study [22]. Then we performed correlation analysis on gene and pathway levels between BAS from the previous study [22] and our metrics (Figure 12). We found positive correlations between BAS and our mutation metrics both on gene and pathway levels, but no correlation was found with the expression data (Figure 12). 

**Figure 13:**
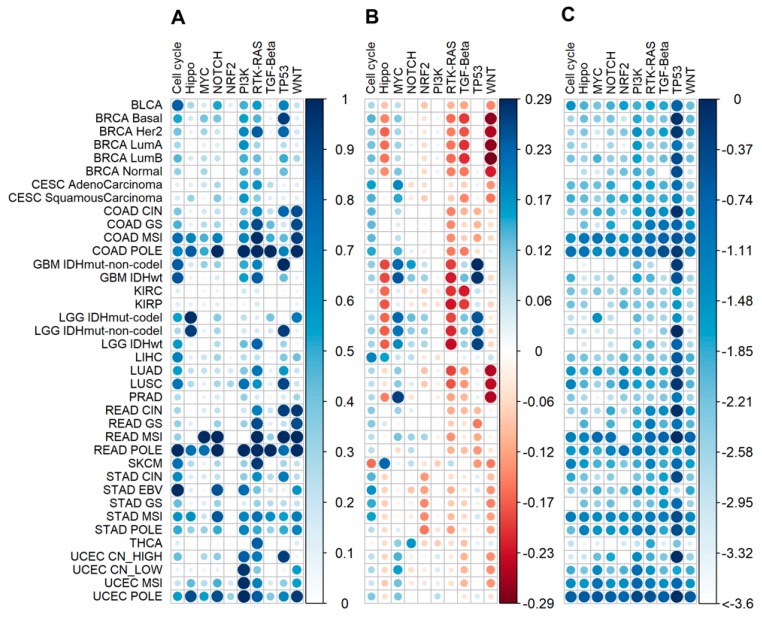
Comparison of pathway functional metrics for 4382 tumor samples common for this and previous study [22] calculated for ten signaling pathways from [22]. (**A**) Average BAS (on pathway level) per tumor subtype. (**B**) Average PAL per tumor subtype. (**C**) Average lg(PI) per tumor subtype. Molecular tumor subtypes were referred according to [22]: BLCA—Urothelial bladder cancer; BRCA—Breast cancer; CESC—Cervical cancer; KICH—Chromophobe renal cell carcinoma; KIRC—Clear cell kidney carcinoma; COAD—Colon adenocarcinoma; READ—Rectal adenocarcinoma; SKCM—Cutaneous melanoma; GBM—Glioblastoma multiforme; LIHC—Liver hepatocellular carcinoma; LGG—Lower Grade Glioma; LUAD—Lung adenocarcinoma; LUSC—Lung squamous cell carcinoma; KIRP—Papillary kidney carcinoma; PRAD—Prostate adenocarcinoma; STAD—Stomach adenocarcinoma; THCA—Papillary thyroid carcinoma; UCEC—Uterine corpus endometrial carcinoma; CIN—Chromosomal Instability; CN_HIGH—copy-number high; CN_LOW—copy-number low; EBV—Epstein-Barr Virus; GS—Genomically Stable; Her2—Her2-enriched; IDHwt—IDH1-wild-type; IDH mutant-codel—*IDH* mutant with codeletion of chromosome arm 1p and 19q; IDH mutant-non-codel—*IDH* mutant with euploid 1p/19q; LumA—Luminal A; LumB—Luminal B; MSI—Microsatellite Instability; POLE—polymerase ε mutant subtype.

We also compared our results with the trends previously revealed for BAS in different tumor subtypes. To this end, we calculated on the pathway level average BAS, PAL and PI scores per tumor subtype (Figure 13). At the gene level, we compared average BAS, CNR and nMR per tumor subtype (Supplementary File S2). Overall, we observed similarities for the mutational scores, but no common trends for the comparison with the expression data. Thus, previously reported complex binary alteration score partially resembles to mutation metrics like nMR ad PI because BAS includes mutation data as compound, but it doesn’t reflect changes at the expression level (Figure 12 and Figure 13).

Totally, in our study we analyzed 4910 individual cancer gene expression and matching whole exome sequencing profiles collectively covering ~1.3 million mutations in thirteen cancer types. We found that for both mutation and gene expression data, the intertumoral similarities were much higher at the pathway level than at the level of individual genes. We identified common trends for the representatives of *signaling/cytoskeleton* and *metabolism/DNA repair* groups of pathways at the level of mutation data. Most importantly, the *signaling/cytoskeleton* group members were outstandingly enriched by the most highly mutated genes and deficient by the genes with the low mutation levels. This suggests their initiator roles in carcinogenesis and the auxiliary/supporting roles for the other groups of molecular pathways. 

However, at the level of gene expression, the *DNA repair* group showed markedly upregulated activation levels in cancers, whereas for the other groups of pathways, the downregulated members prevailed. On the other hand, DNA repair pathways also demonstrated the most congruent activation patterns among all the tumor samples.

Our results also confirmed largely downregulated activities of the *metabolic* molecular pathways in cancer. In many reports, a focus was made on the metabolic alterations in tumors, so that cancer is even called a metabolic disorder [65,66,67,68]. We identified here multiple differentially regulated/mutated metabolic pathways in cancer, and several related biochemical processes were suggested by the gene ontology analyses. We found that metabolic pathways had the lowest mutational burden, and revealed that only the pathways related to nucleotide metabolism were significantly systemically up-regulated that argues some previous reports about hyperactivation of common metabolic background [65,66,67,68].

More specifically, the least mutated pathways dealt (*cytoskeleton*) with membrane organization, receptor-mediated endocytosis; (*DNA repair*) with regulation of DNA quality and associated DNA repair, and with rearrangement of immunoglobulin loci; (*metabolic*) with biosynthesis of nucleotides and DNA replication; (*signaling*) with negative regulation of glucocorticoid receptor pathway, regulation of hormone levels, circadian rhythms, regulation of H3 histone deacetylation and protein catabolism. 

In turn, the most strongly mutated pathways were responsible for (*cytoskeleton*) exocytosis, cell cycle progression, processing and presentation of antigens via MHC class II; (*DNA repair*) cellular response to stress, chromosome organization, hydrolysis of phosphodiester bonds; (*metabolic*) drug response and catabolism, arachidonic acid secretion, fatty acid derivative pathways, glycosylation, sulfur and benzene-containing compound pathways, production of mitochondrial RNA, and tRNA aminoacetylation for protein translation; (*signaling*) regulation through phosphatidyl inositol, cell surface receptor pathways, growth and proliferation, transport of calcium, transcription by RNA polymerase II, and regulation of immune cell activation. On the other hand, the most strongly cancer- downregulated pathways related to (*cytoskeleton*) movement of cell or subcellular components and locomotion, regulation of development and cell proliferation; (*metabolic*) cellular ketone body metabolism, neurotransmitter catabolic processes, sulfur pathways, drug response pathways; (*signaling*) transcription by RNA polymerase II, interleukin-7-mediated pathway.

Among the most strongly cancer-upregulated pathways, there were no *cytoskeleton* pathways, whereas the others dealt with (*DNA repair*) rearrangement of immune receptor loci, protein localization on chromosomes and chromosomal organization; (*metabolic*) oligosaccharide biosynthesis, nucleotide biosynthesis and DNA replication; (*signaling*) cell migration and adhesion, regulation of cell death, calcium ion transmembrane transport, extracellular matrix organization, regulation of mRNA stability, processing and presentation of exogenous peptide antigen via MHC class I, and activation of immune cells.

Finally, our study poses a challenge of analyzing the whole cancer interactome model where separate pathways would be connected into a single graph with known connectivity and functional relationships between its nodes. In the future, this type of analysis could be beneficial for personalized finding of causative cancer mutations and clinically actionable therapeutic molecular targets.

## 4. Materials and Methods

### 4.1. Mutation Data

DNA mutation data were extracted from the Catalogue Of Somatic Mutations In Cancer (COSMIC) project database [69]. We used genome-wide screen dataset, including whole exome and genome sequencing data, database version 76. The whole dataset included information for 19 434 tumor samples of different localizations obtained from different sources but here we considered only the samples obtained from The Cancer Genome Atlas (TCGA) project because of their uniform experimental and analytic pipeline [42]. We took only tumor samples that had the matching RNA sequencing data for the same tumor samples and also had corresponding RNA sequencing data for the normal samples of the same tissue type on Genomic Data Commons (GDC) data portal [70]. We extracted non-synonymous somatic mutations by selecting the following mutation types: “substitution-missense”, “deletion-frameshift”, “substitution-nonsense”, “insertion-in frame”, “deletion-in frame”, “insertion-frameshift”, “complex-deletion in frame”, “nonstop extension”, “complex”, “complex-compound substitution”, “complex-frameshift”, “complex-insertion in frame” (Supplementary Table S10). The final dataset contained 1,252,669 mutation records for 19,608 genes from 4910 individual tumor samples. The tumor samples selected had the following primary localizations: bladder, breast, brain, cervix, kidney, colon and rectum, liver, lung, prostate, skin, stomach, thyroid and uterus (Supplementary Table S5). Every patient case corresponded to only one tumor sample. Individual mutation profiles are listed in Supplementary Table S11.

### 4.2. Gene Expression Data

Gene expression profiles were obtained from GDC data portal [70]. We downloaded RNA sequencing data (HTSeq counts modification) for the tumor samples that also had somatic mutation profiles from COSMIC database [48]; the normal tissues were taken for the same tissue types as the tumor samples investigated. Totally, gene expression data were collected for 655 normal and 4910 tumor tissue samples (13 cancer types, Supplementary Table S5). Individual gene expression profile IDs are listed in Supplementary Table S12. 

### 4.3. Pathway Databases

The gene structures and molecular architectures of 419 intracellular pathways were extracted from the public databases Reactome [43], NCI Pathway Interaction Database [44], Kyoto Encyclopedia of Genes and Genomes [17], HumanCyc [45], Biocarta [46], Bio-rad [71], Qiagen [47] (Supplementary Table S1). 

To increase statistical accuracy, we considered only 419 molecular pathways including ten or more genes. These pathways were classified into four functional groups by expert supervision: 278 signaling, 72 metabolic, 48 DNA repair and 47 cytoskeleton pathways (Supplementary Table S1; gene lists are available in Supplementary Table S2). 26 pathways were marked as simultaneously signaling and DNA repair pathways. 

### 4.4. Calculation of Mutation Frequency Metrics

*Pathway instability* (*PI*) reflects mutation enrichment of a molecular pathway [14]. *PI* calculation utilizes mutation frequencies of genes forming a pathway. To assess mutation frequencies of *individual genes*, we introduced *normalized mutation rate* (*nMR*) value expressed by the formula:

(1) $$nMR_{n}=\frac{N\;mut\;\left(n,g\right)\times 1000}{N\;samples\;\left(g\right)\times Length\;CDS\;\left(n\right)}$$

where *nMR_n_* is *normalized mutation rate* of gene *n*; *N mut*(*n*,*g*) is total number of mutations for gene *n* in group of samples *g*; *N samples* (*g*) is number of samples in group *g*; *Length CDS* (*n*) is length of coding DNA sequence (CDS) of gene *n* in nucleotides. *PI* levels were calculated as follows:

(2) $$PI_{p}=\frac{\sum_{n}nMR_{n}PG_{p,n}}{N_{p}}$$

where *PI_p_* is *PI* for pathway *p*; *nMR_n_* is as described above; $PG_{p.n}$ is pathway-gene indicator that equals to one if gene *n* belongs to pathway *p*, or equals to zero if not; $N_{p}$—total number of gene products belonging to pathway *p.* The calculations were made using Amazon and Microsoft Azure cloud services.

### 4.5. Calculation of Pathway Activation Level

*Pathway activation level (PAL)* characterizes cumulative changes of expression levels of genes belonging to a certain molecular pathway [12,23,50,72,73]. PAL is calculated as follows [23]:

(3) $$PAL_{p}=\underset{n}{\sum }ARR_{np}*\mathrm{lg}\left(CNR_{n}\right)/\underset{n}{\sum }|ARR_{np}|$$

where *PAL_p_* is *PAL* for pathway p, *CNR_n_* is *case-to-normal ratio*, the ratio of gene *n* expression level in a tumor sample under study to an average level for the control group; *ARR* (*activator/repressor role*) is Boolean flag that depends on function of gene n product in pathway p. ARR is −1 if gene product *n* inhibits pathway *p*; 1 if *n* activates apthway; 0 if *n* has ambiguous or unclear role in a pathway; 0.5 or −0.5, if *n* is rather activator of a pathway or its inhibitor, respectively. The calculations were made using Amazon and Microsoft Azure cloud services.

### 4.6. Gene Ontology Analysis

We performed analysis of biological processes involving gene sets under investigation using GOrilla tool for Gene Ontology terms analysis [52]. We used modification of two unranked gene lists: *target dataset* was the gene set of interest and *background* was list of protein coding human genes from Human Genome Organisation (HUGO) Gene Nomenclature Committee (HGNC) database, version 20170717 [74], *p*-value threshold was set as 10^−3^. 

Gorilla outputs were visualized using REVIGO viewer with the default settings (*allowed similarity* = 0.7, semantic similarity measure was SimRel), except for dispensability cut-off level (0.25). We excluded from visualization the non-informative general terms like “biological processes” and irrelevant terms like “kidney morphogenesis” by expert curation. 

### 4.7. Comparison with the Study “Oncogenic Signaling Pathways in The Cancer Genome Atlas”

We used Genomic Alteration matrixes of gene and pathway levels from the study “Oncogenic Signaling Pathways in The Cancer Genome Atlas” [22] to compare the results of this study. Genomic Alteration matrixes contained information about the following alterations: copy-number alterations, mutations, fusions or epigenetic silencing in the form of binary alteration score (BAS). If one or more alterations was in the gene in the tumor sample, BAS (gene level) for this gene and this tumor sample was “1”, otherwise—“0”. If one or more genes had BAS equal to one in the pathway in the tumor sample, BAS (pathway level) for this pathway and this tumor sample was “1”, otherwise—“0”. We used BAS for ten signaling pathways: (1) cell cycle, (2) Hippo signaling, (3) Myc signaling, (4) Notch signaling, (5) oxidative stress response/ Nuclear factor erythroid 2-related factor 2 (NRF2) pathway, (6) PI-3-Kinase signaling, (7) receptor-tyrosine kinase (RTK)/ Rat sarcoma (RAS) kinase/ Mitogen-Activated Protein (MAP) kinase signaling, (8) Transforming growth factor beta (TGFβ) signaling, (9) P53 and (10) β-catenin/ Wingless-related integration site (WNT) signaling, and 186 enclosed genes [22]. 

The study [22] analyzed 9125 tumor samples of 64 tumor subtypes, among them 4382 (representing 38 tumor subtypes) were also included in our study. This dataset of 4382 samples of 38 tumor subtypes was used for the comparative analysis. Tumor sample barcodes and molecular subtypes are given in Supplementary Table S13. For ten above pathways, we calculated expression and mutation functional metrics: PAL, PI and CNR, nMR for 186 genes (Supplementary Table S14). Average BAS on gene level, BAS on pathway level, CNR, nMR, PAL and PI per tumor subtype were calculated as arithmetic mean for tumor samples of a tumor subtype under consideration. 

## 5. Conclusions

Using high throughput mutation and gene expression data, we established pathway activation and mutation enrichment metrics of 278 signaling, 72 metabolic, 48 DNA repair and 47 cytoskeleton molecular pathways in 4910 individual cancer samples RNA sequencing and whole exome sequencing profiles representing thirteen cancer types. We found no correlation between mutation enrichment and expression changes both at the individual gene and molecular pathway levels. We observed generally congruent pathway activation profiles for different cancer types. The highest cancer-specific activation levels were seen in the DNA repair pathways, which had also the highest mutation enrichment levels. Moreover, the DNA repair pathways also demonstrated the most congruent activation patterns among all the tumor samples. However, the signaling and cytoskeleton pathways had the highest proportions of outstandingly frequently mutated genes thus suggesting their initiator roles in carcinogenesis and the supporting roles for the other groups of molecular pathways.

## Figures and Tables

**Table 1:** Similarity coefficients between gene compositions of different groups of molecular pathways. Jaccard coefficients are shown above and Szymkiewicz-Simpson coefficients—below the main diagonal.

Intersection	Signaling	Metabolic	Cytoskeleton	DNA Repair
Signaling	1	0.019	0.196	0.119
Metabolic	0.087	1	0.014	0.006
Cytoskeleton	0.621	0.031	1	0.045
DNA repair	0.738	0.016	0.135	1

**Table 2:** Percentage of 4910 tumor samples with mutated genes of four pathway groups.

Minimal Number of Mutated Genes	Cytoskelton	DNA Repair	Metabolic	Signaling	Minimal Number of Mutated Genes	Cytoskelton	DNA Repair	Metabolic	Signaling
1	78%	53%	58%	94%	11	17%	6%	9%	46%
2	65%	35%	41%	89%	12	16%	5%	8%	42%
3	54%	24%	31%	84%	13	15%	5%	8%	39%
4	45%	18%	24%	78%	14	14%	4%	7%	37%
5	38%	14%	19%	72%	15	13%	4%	6%	35%
6	32%	11%	16%	66%	16	12%	4%	6%	33%
7	27%	9%	14%	61%	17	11%	3%	5%	31%
8	24%	8%	12%	56%	18	10%	3%	5%	29%
9	21%	7%	11%	52%	19	10%	3%	5%	28%
10	19%	6%	10%	48%	20	9%	3%	5%	26%

## Supplementary Materials

The following are available online at https://www.mdpi.com/2072-6694/12/2/271/s1, Table S1: Referenced list of molecular pathways investigated in this study, Table S2: Gene lists of pathway groups: signaling, metabolic, DNA repair, cytoskeleton pathways, Table S3: Averaged normalized mutation rates (nMRs) for 19 608 genes, for 4910 cancer samples, Table S4: Averaged normalized mutation rates (nMRs) for 19 608 genes, given separately for 13 cancer types, Table S5: Structure of dataset of 4910 cancer samples and 655 normal samples, IDs of expression and mutation data samples, Table S6: PAL scores for 4910 tumor samples, Table S7: PI scores for 4910 tumor samples, Table S8: Top and bottom 15% frequently mutated pathways and their gene compositions, Table S9: Top and bottom cancer-upregulated pathways and their gene compositions, Table S10: Mutation types in cancers, Table S11: Individual mutation profiles of 4910 tumor samples, Table S12: Links to individual gene expression profiles of 4910 cancer samples and 661 matched normal samples, Table S13: Barcodes and molecular subtypes of 4832 tumor samples, which were included in our study and in [22], Table S14: Expression and mutation functional metrics: PAL, PI for ten signaling pathways and CNR, nMR for 186 genes, for 4382 tumor samples. Supplementary File S1: Cancer type-specific distributions of pairwise Spearman correlation coefficients between all possible combinations of tumor samples under investigation for mutation and expression data at both gene wise (nMR, CNR) and pathway (PAL, PI) level in cytoskeleton, DNA repair, metabolic and signaling pathway groups. Vertical dashed lines indicate the medians of the distributions, Supplementary File S2: Comparison of gene functional metrics for 4382 tumor samples common for this and previous study [22] calculated for 186 genes from [22]: Average BAS (on gene level), average CNR, average lg(nMR) per tumor subtype. Molecular tumor subtypes were referred according to [22]: BLCA—Urothelial bladder cancer; BRCA—Breast cancer; CESC—Cervical cancer; KICH—Chromophobe renal cell carcinoma; KIRC—Clear cell kidney carcinoma; COAD—Colon adenocarcinoma; READ—Rectal adenocarcinoma; SKCM—Cutaneous melanoma; GBM—Glioblastoma multiforme; LIHC—Liver hepatocellular carcinoma; LGG—Lower Grade Glioma; LUAD—Lung adenocarcinoma; LUSC—Lung squamous cell carcinoma; KIRP—Papillary kidney carcinoma; PRAD—Prostate adenocarcinoma; STAD—Stomach adenocarcinoma; THCA—Papillary thyroid carcinoma; UCEC—Uterine corpus endometrial carcinoma; CIN—Chromosomal Instability; CN_HIGH—copy-number high; CN_LOW—copy-number low; EBV—Epstein-Barr Virus; GS—Genomically Stable; Her2—Her2-enriched; IDHwt—IDH1-wild-type; IDH mutant-codel—*IDH* mutant with codeletion of chromosome arm 1p and 19q; IDH mutant-non-codel—*IDH* mutant with euploid 1p/19q; LumA—Luminal A; LumB—Luminal B; MSI—Microsatellite Instability; POLE—polymerase ε mutant subtype. Supplementary Figure S1. Correlation of nMR and CNR values for 4910 cancer samples under investigation.
