# Supplementary material for: Mutation Enrichment and Transcriptomic Activation Signatures of 419 Molecular Pathways in Cancer
Source: Cancers (Basel). 2020 Jan 22;12(2):271. doi: 10.3390/cancers12020271 (PMC7073226; doi:10.3390/cancers12020271)
Supplement: Supplementary file 1 [file cancers-12-00271-s001.zip › supplementary revised/supplementary legend.docx]

**Supplementary Materials**


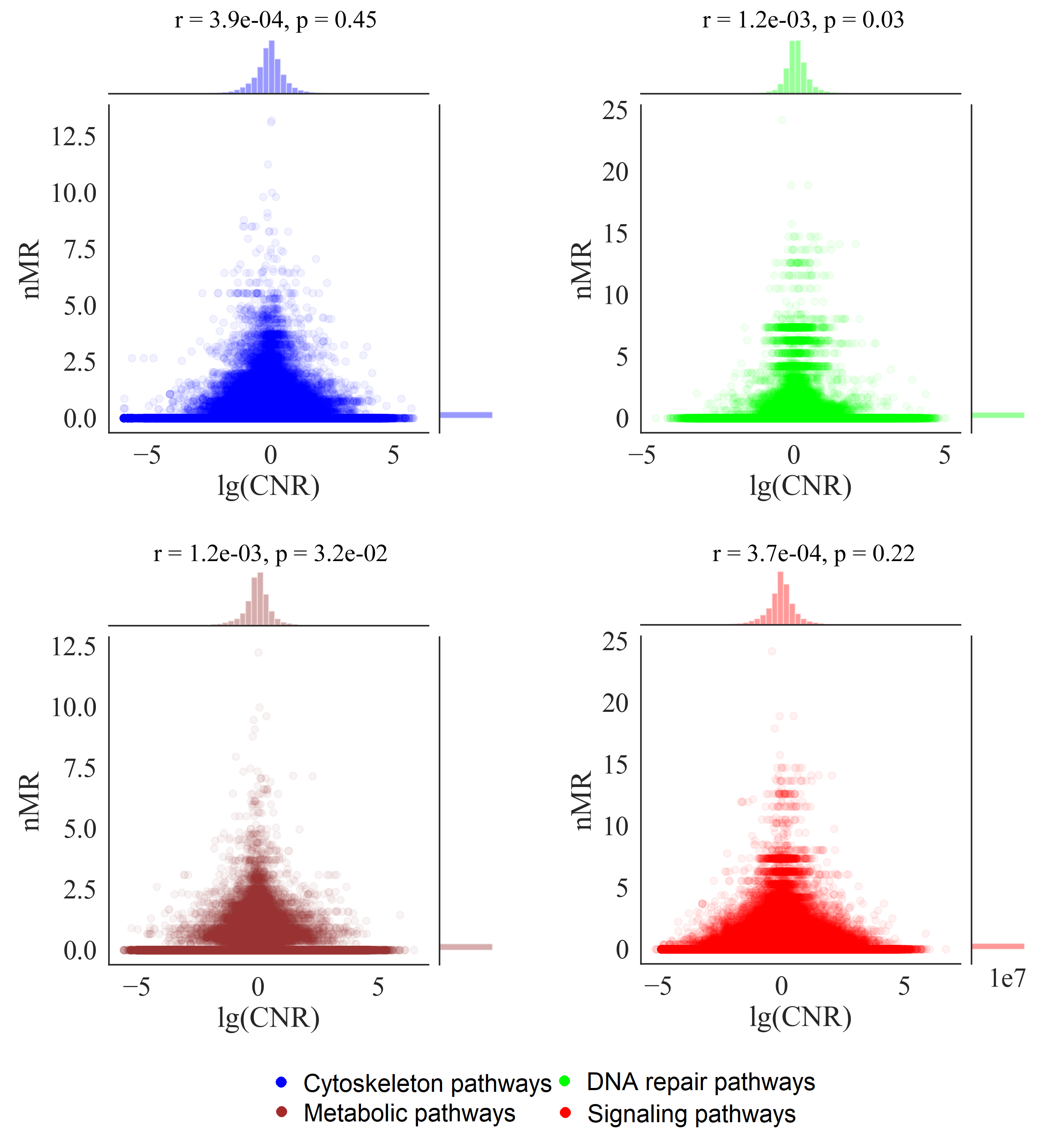


**Figure S1.** Correlation of nMR and CNR values for 4 910 cancer samples under investigation.

**Table S1.** Referenced list of molecular pathways investigated in this study.

**Table S2.** Gene lists of pathway groups: signaling, metabolic, DNA repair, cytoskeleton pathways.

**Table S3.** Averaged normalized mutation rates (nMRs) for 19 608 genes, for 4 910 cancer samples.

**Table S4.** Averaged normalized mutation rates (nMRs) for 19 608 genes, given separately for 13 cancer types.

**Table S5.** Structure of dataset of 4 910 cancer samples and 655 normal samples, IDs of expression and mutation data samples.

**Table S6.** PAL scores for 4 910 tumor samples.

**Table S7.** PI scores for 4 910 tumor samples.

**Table S8.** Top and bottom 15% frequently mutated pathways and their gene compositions.

**Table S9.** Top and bottom cancer-upregulated pathways and their gene compositions

**Table S10.** Mutation types in cancers.

**Table S11.** Individual mutation profiles of 4 910 tumor samples.

**Table S12.** Links to individual gene expression profiles of 4910 cancer samples and 661 matched normal samples.

**Table S13.** Barcodes and molecular subtypes of 4832 tumor samples, which were included in our study and in [22].

**Table S14.** Expression and mutation functional metrics: PAL, PI for ten signaling pathways and CNR, nMR for 186 genes, for 4382 tumor samples.

**Supplementary File 1.** Cancer type-specific distributions of pairwise Spearman correlation coefficients between all possible combinations of tumor samples under investigation for mutation and expression data at both gene wise (nMR, CNR) and pathway (PAL, PI) level in cytoskeleton, DNA repair, metabolic and signaling pathway groups. Vertical dashed lines indicate the medians of the distributions.

**Supplementary File 2.** Comparison of gene functional metrics for 4382 tumor samples common for this and previous study [22] calculated for 186 genes from [22]: Average BAS (on gene level), average CNR, average lg(nMR) per tumor subtype. Molecular tumor subtypes were referred according to [22]: BLCA - Urothelial bladder cancer; BRCA - Breast cancer; CESC - Cervical cancer; KICH - Chromophobe renal cell carcinoma; KIRC - Clear cell kidney carcinoma; COAD - Colon adenocarcinoma; READ - Rectal adenocarcinoma; SKCM - Cutaneous melanoma; GBM - Glioblastoma multiforme; LIHC - Liver hepatocellular carcinoma; LGG - Lower Grade Glioma; LUAD - Lung adenocarcinoma; LUSC - Lung squamous cell carcinoma; KIRP - Papillary kidney carcinoma; PRAD – Prostate adenocarcinoma; STAD - Stomach adenocarcinoma; THCA - Papillary thyroid carcinoma; UCEC - Uterine corpus endometrial carcinoma; CIN - Chromosomal Instability; CN_HIGH – copy-number high; CN_LOW – copy-number low; EBV - Epstein-Barr Virus; GS - Genomically Stable; Her2 - Her2-enriched; IDHwt - IDH1-wild-type; IDH mutant-codel - *IDH* mutant with codeletion of chromosome arm 1p and 19q; IDH mutant-non-codel - *IDH* mutant with euploid 1p/19q; LumA - Luminal A; LumB - Luminal B; MSI - Microsatellite Instability; POLE - polymerase ε mutant subtype.
