# Supplementary material for: Mutation Enrichment and Transcriptomic Activation Signatures of 419 Molecular Pathways in Cancer
Source: Cancers (Basel). 2020 Jan 22;12(2):271. doi: 10.3390/cancers12020271 (PMC7073226; doi:10.3390/cancers12020271)
Supplement: Supplementary file 1 [file cancers-12-00271-s001.zip › supplementary revised/Supplementary_File1.pdf]

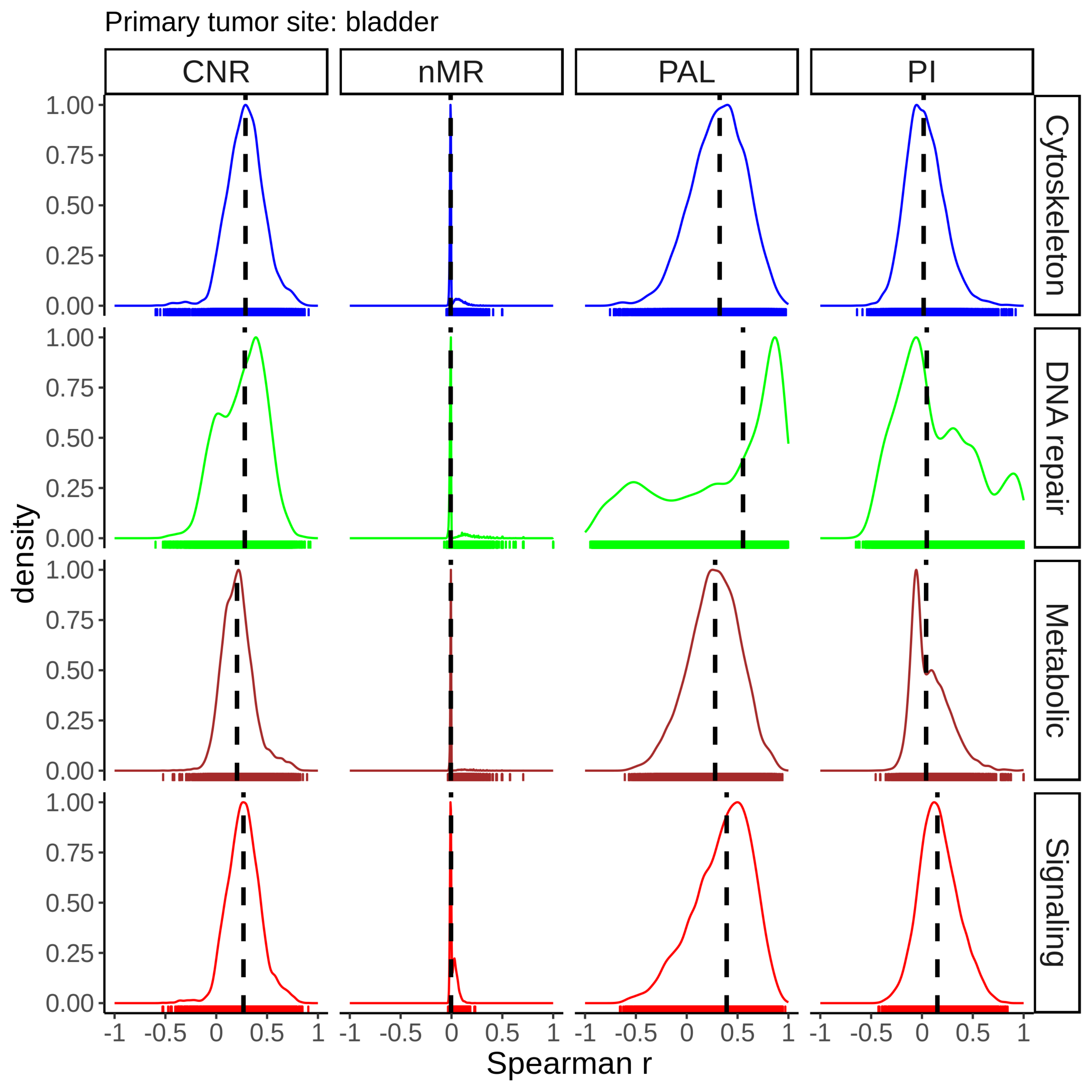

Primary tumor site: bladder
CNR
nMR
PAL
PI
Cytoskeleton
DNA repair
Metabolic
Signaling
density
1.00
0.75
0.50
0.25
0.00
-1
-0.5
0
0.5
1
Spearman r

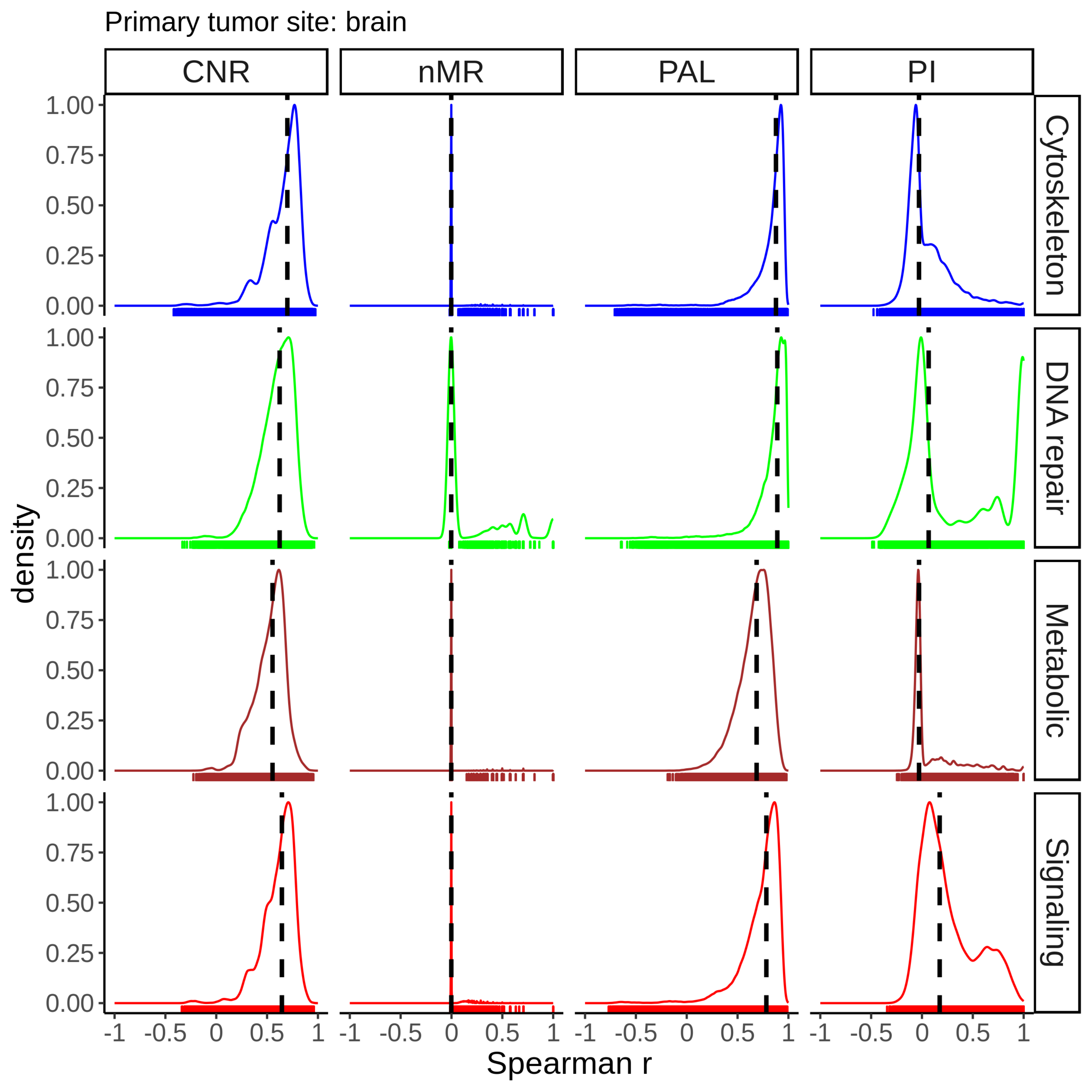

Primary tumor site: brain
CNR
nMR
PAL
PI
Cytoskeleton
DNA repair
Metabolic
Signaling
density
1.00
0.75
0.50
0.25
0.00
-1
-0.5
0
0.5
1
Spearman r

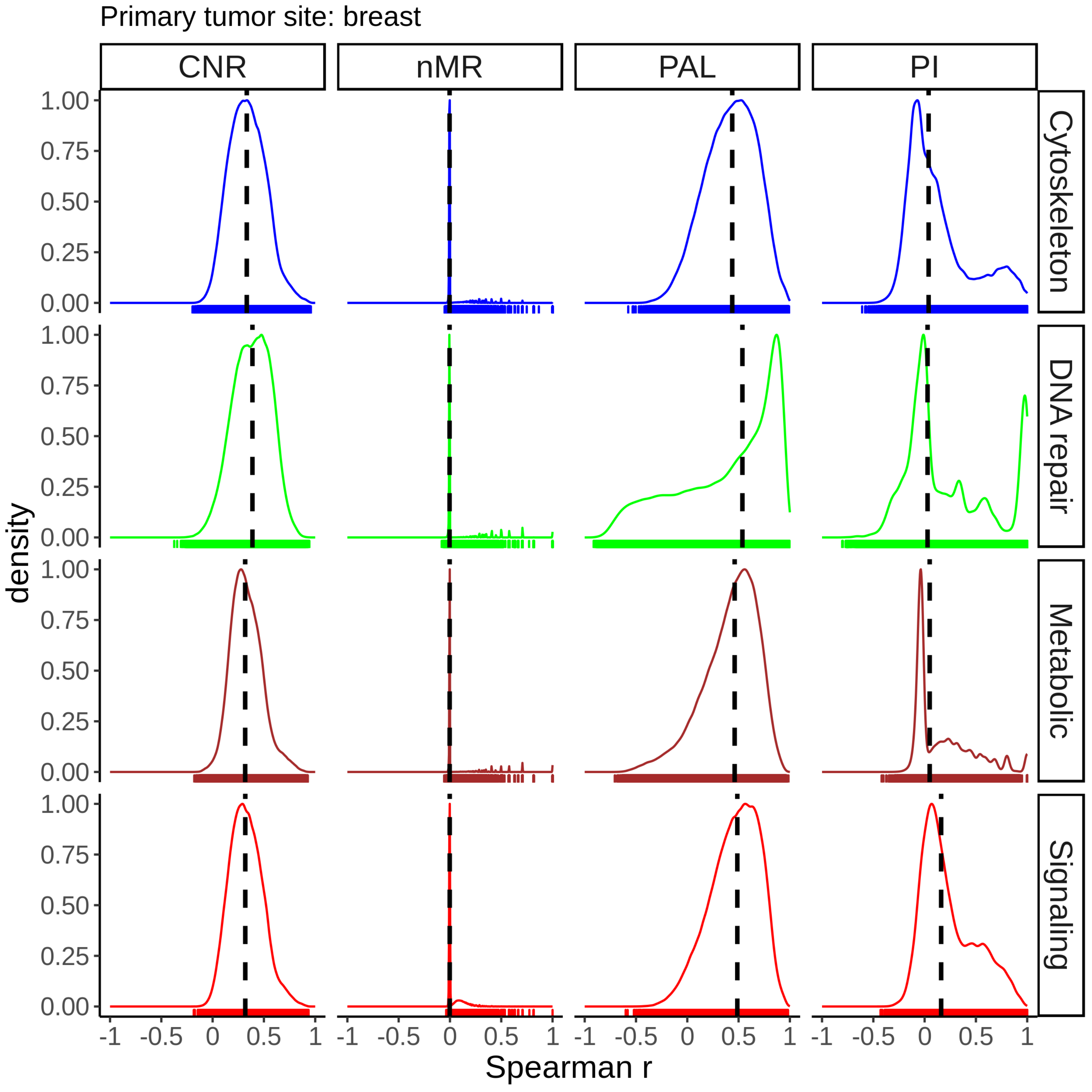

Primary tumor site: breast
CNR
nMR
PAL
PI
Cytoskeleton
DNA repair
Metabolic
Signaling
density
1.00
0.75
0.50
0.25
0.00
-1
-0.5
0
0.5
1
Spearman r

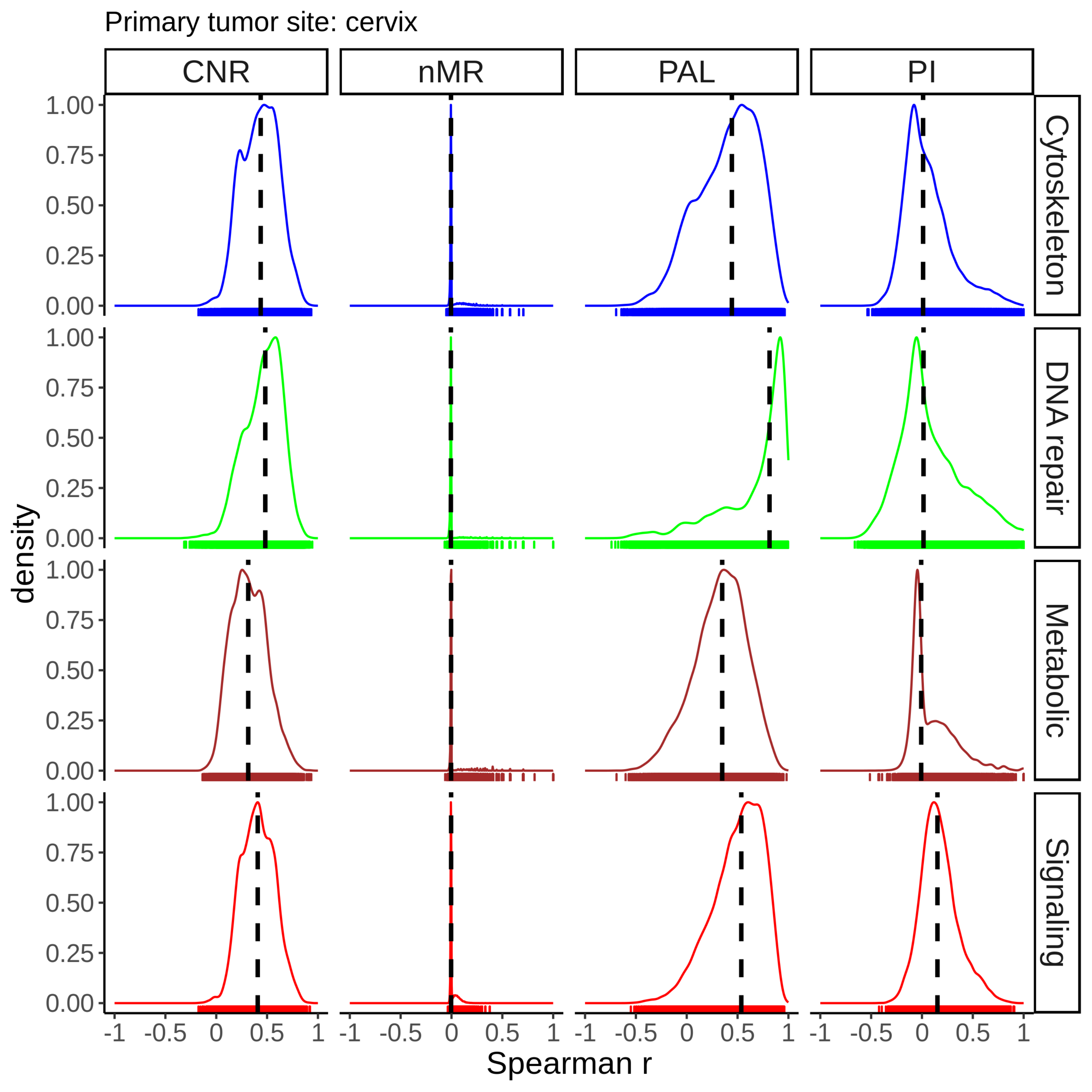

Primary tumor site: cervix
CNR
nMR
PAL
PI
Cytoskeleton
DNA repair
Metabolic
Signaling
density
1.00
0.75
0.50
0.25
0.00
-1
-0.5
0
0.5
1
Spearman r

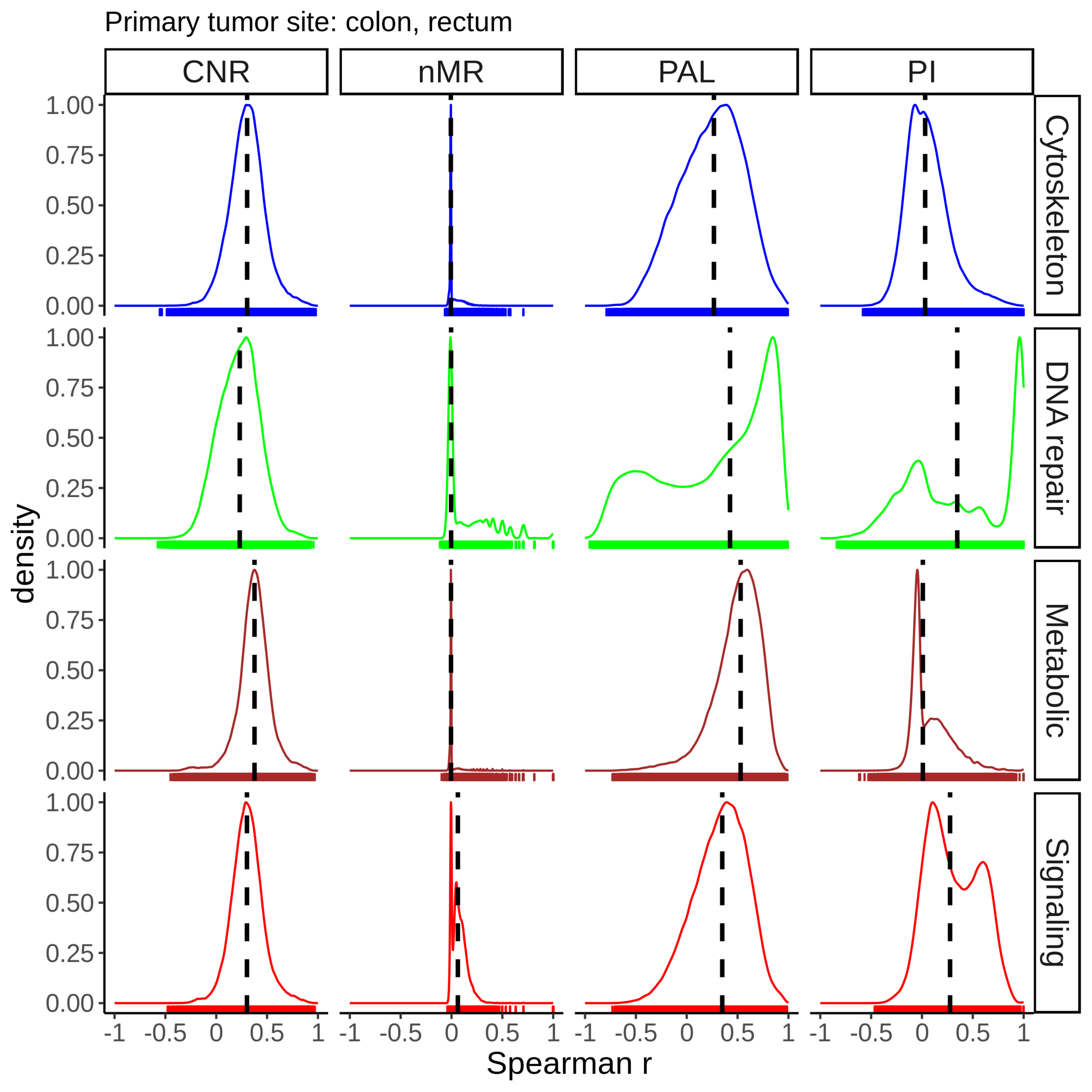
Primary tumor site: colon, rectum
CNR
nMR
PAL
PI
Cytoskeleton
DNA repair
Metabolic
Signaling
density
1.00
0.75
0.50
0.25
0.00
-1
-0.5
0
0.5
1
Spearman r

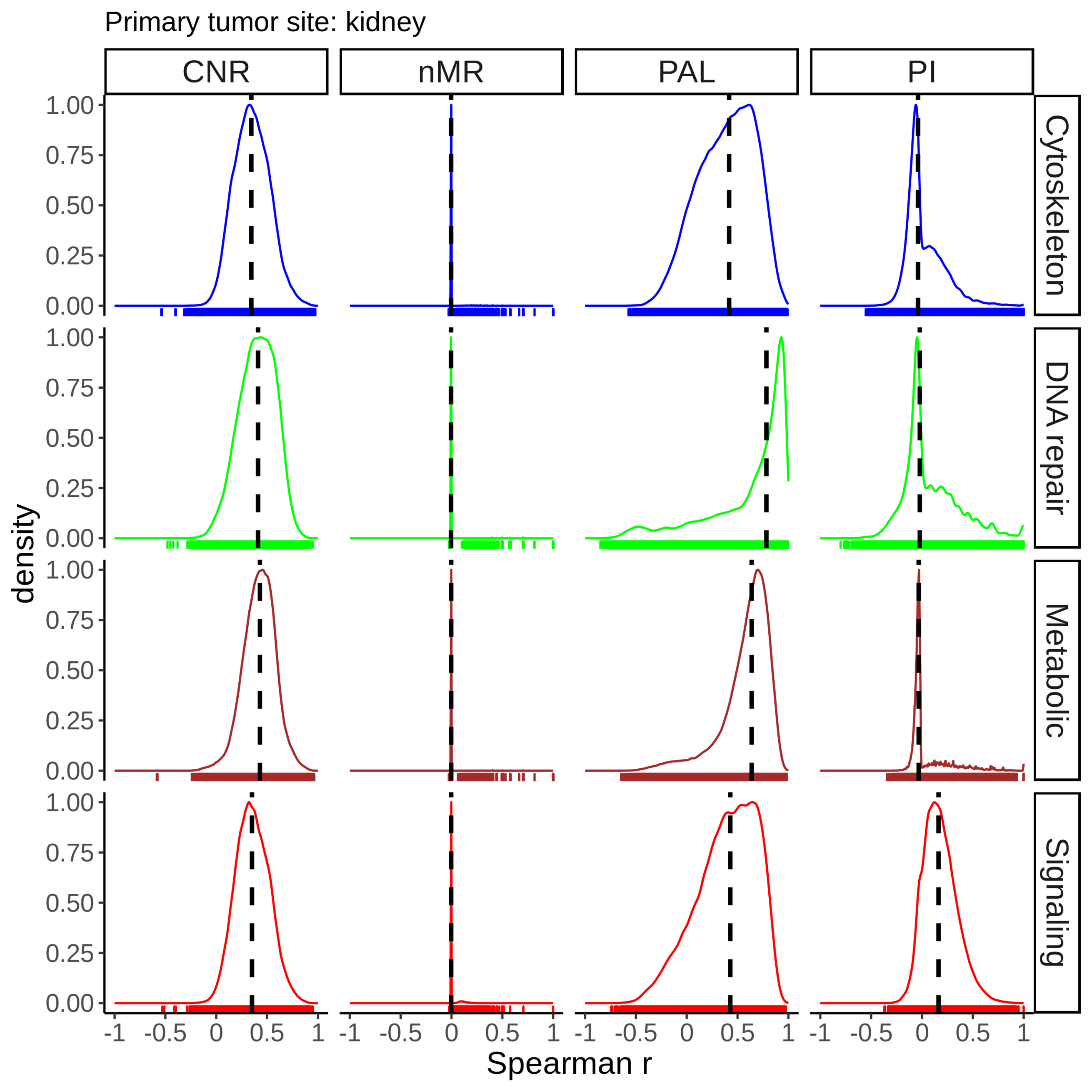

Primary tumor site: kidney
CNR
nMR
PAL
PI
Cytoskeleton
DNA repair
Metabolic
Signaling
1.00
0.75
0.50
0.25
0.00
density
-1
-0.5
0
0.5
1
Spearman r

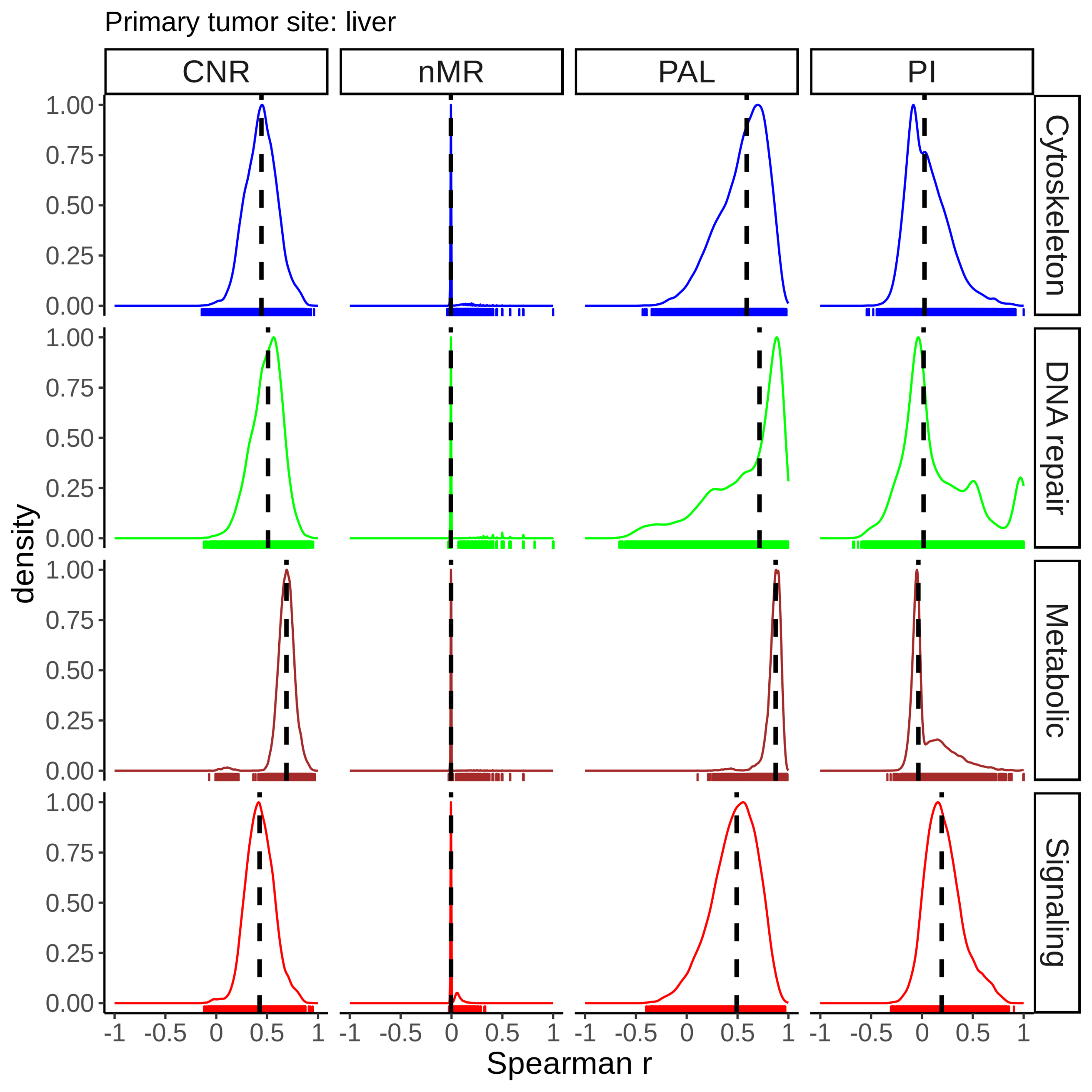
Primary tumor site: liver
CNR
nMR
PAL
PI
Cytoskeleton
DNA repair
Metabolic
Signaling
density
1.00
0.75
0.50
0.25
0.00
-1
-0.5
0
0.5
1
Spearman r

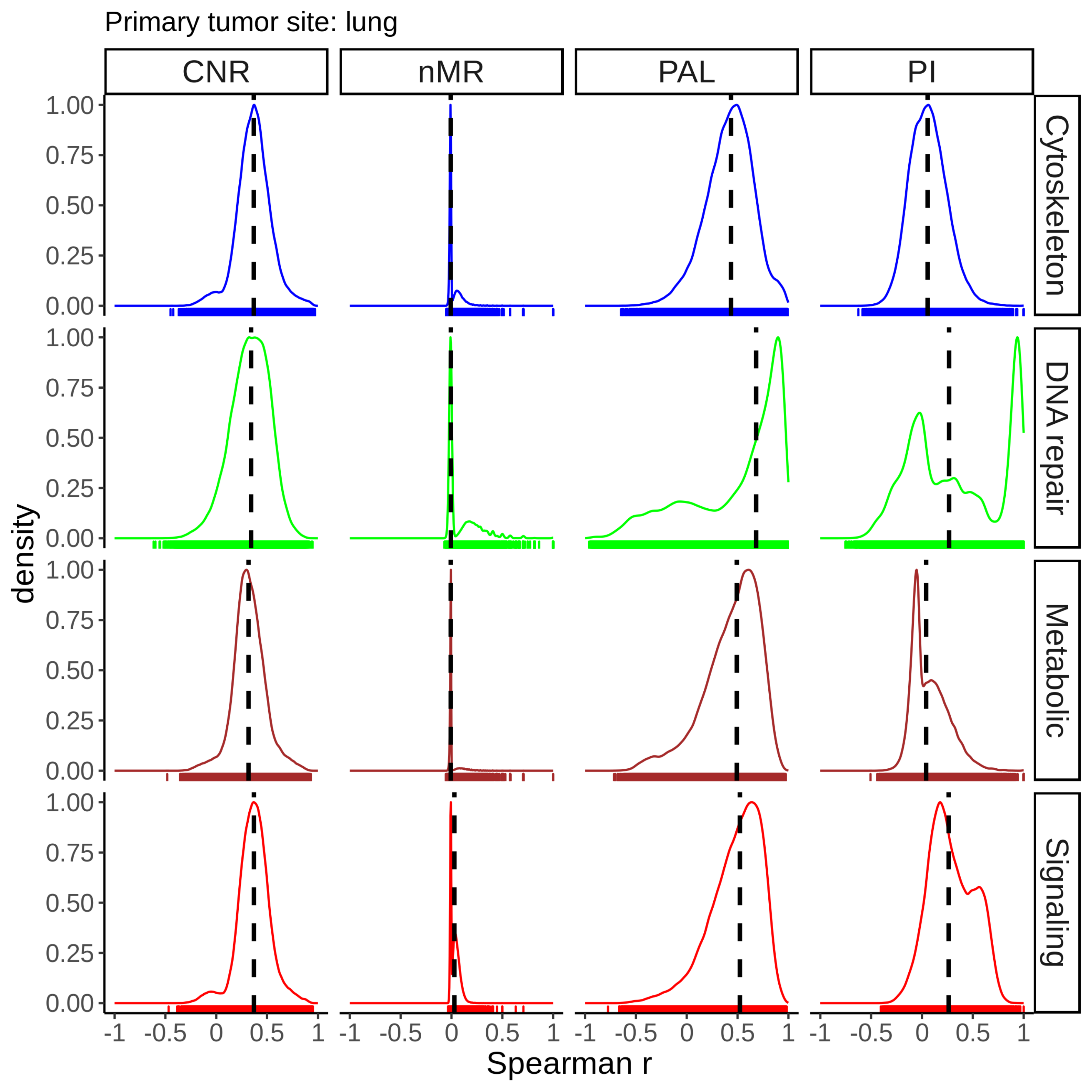

Primary tumor site: lung
CNR
nMR
PAL
PI
Cytoskeleton
DNA repair
Metabolic
Signaling
density
Spearman r
1.00
0.75
0.50
0.25
0.00
-1
-0.5
0
0.5
1

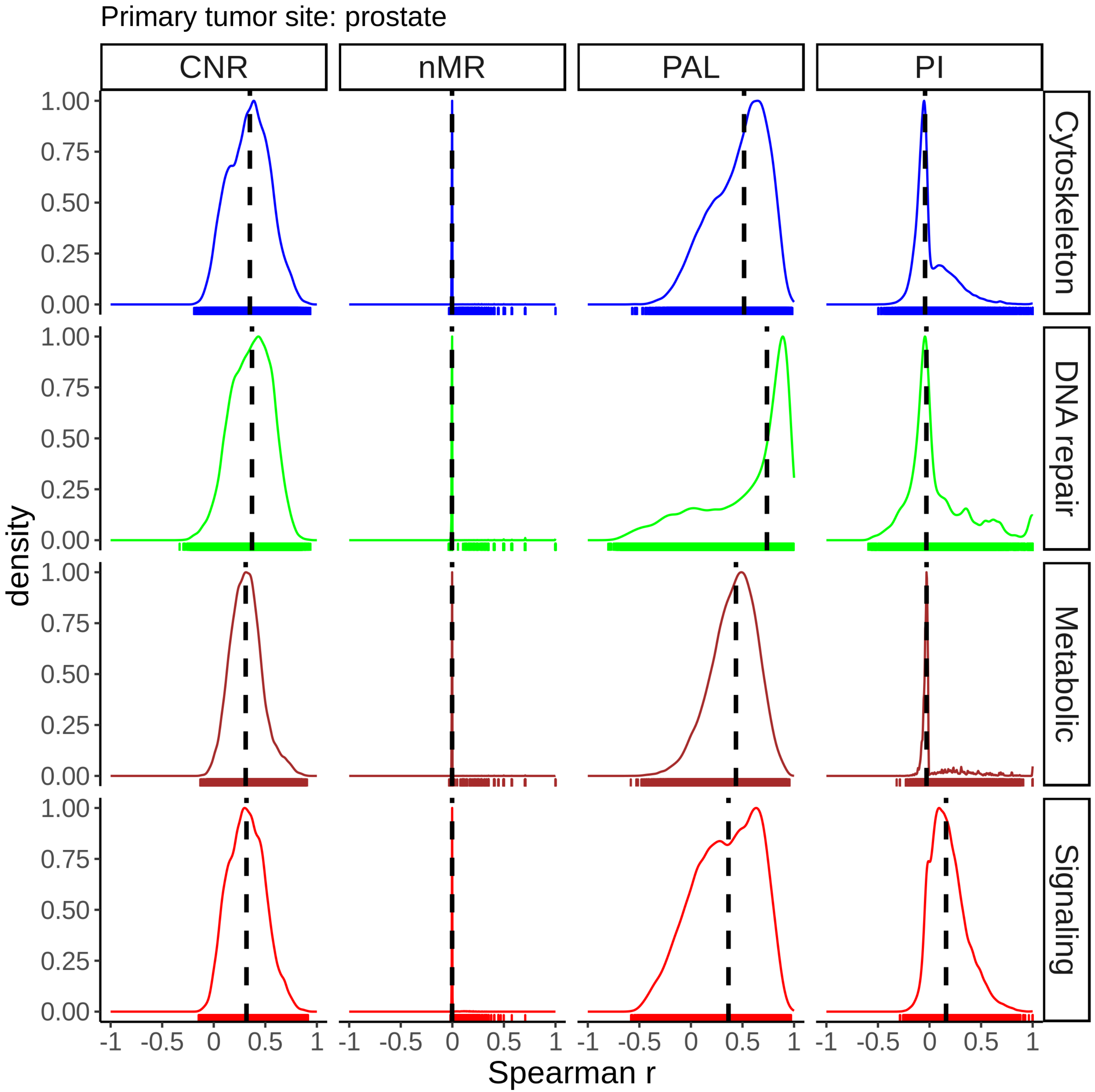
Primary tumor site: prostate
CNR
nMR
PAL
PI
Cytoskeleton
DNA repair
Metabolic
Signaling
density
1.00
0.75
0.50
0.25
0.00
-1
-0.5
0
0.5
1
Spearman r

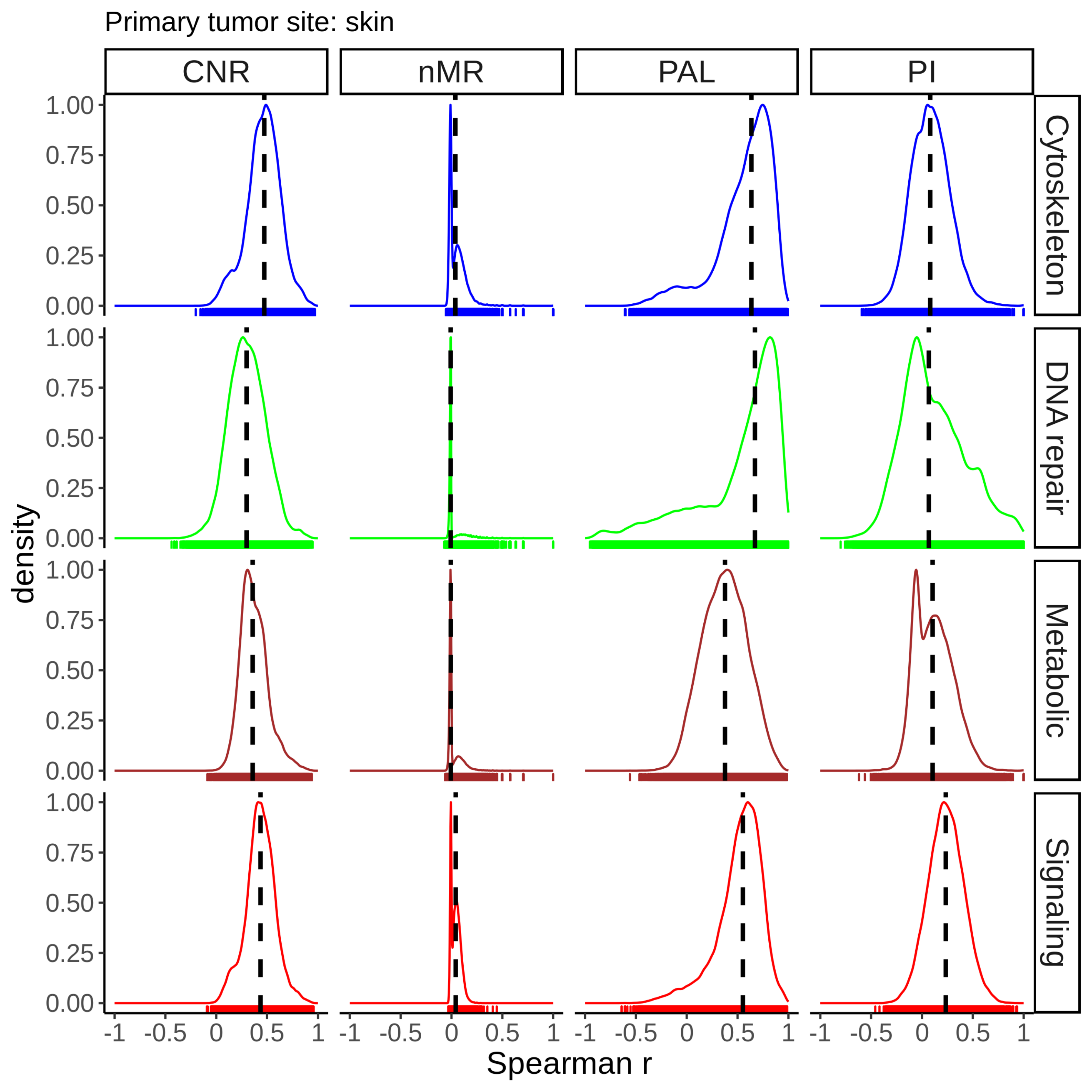

Primary tumor site: skin
CNR
nMR
PAL
PI
Cytoskeleton
DNA repair
Metabolic
Signaling
density
1.00
0.75
0.50
0.25
0.00
-1
-0.5
0
0.5
1
Spearman r

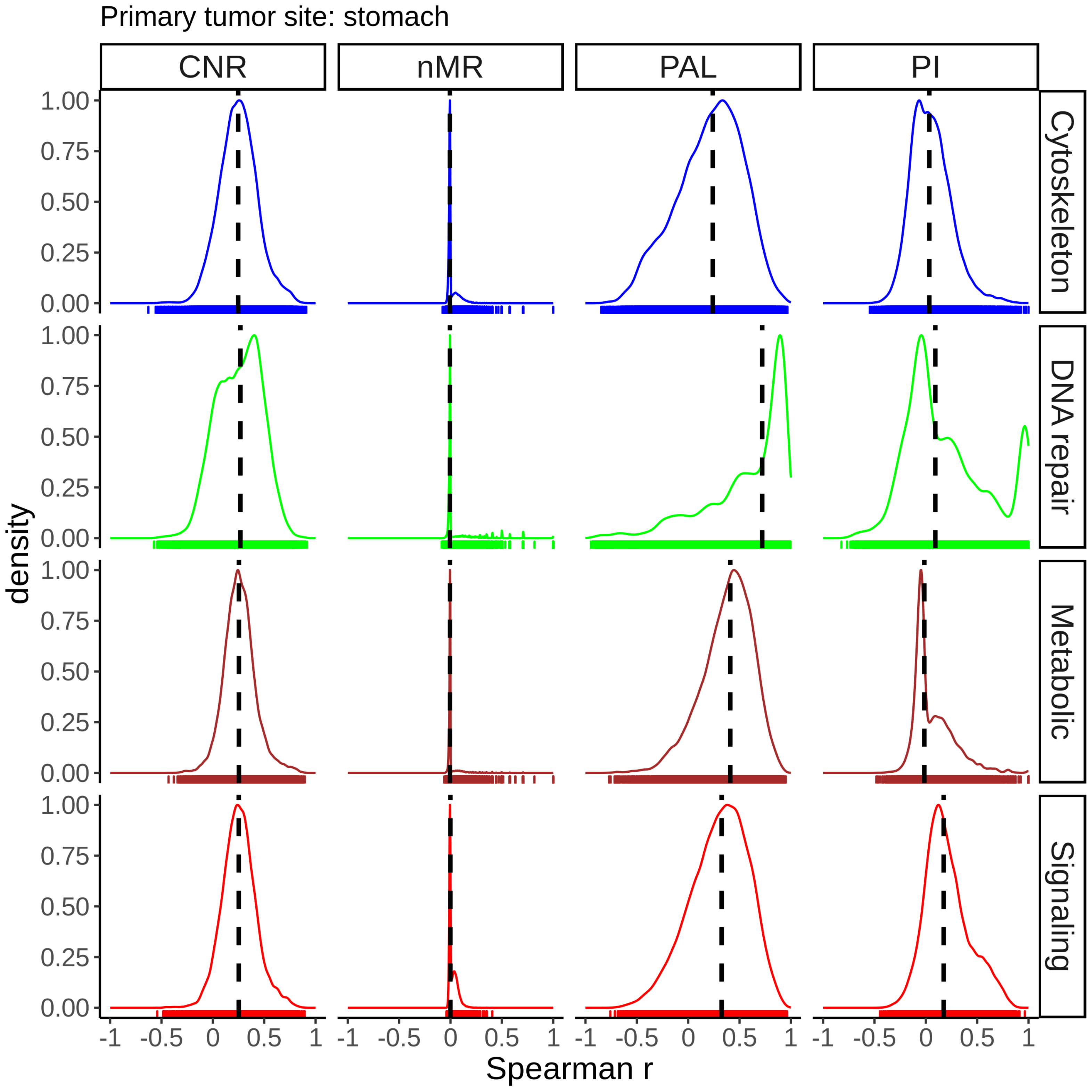

Primary tumor site: stomach
CNR
nMR
PAL
PI
Cytoskeleton
DNA repair
Metabolic
Signaling
density
1.00
0.75
0.50
0.25
0.00
-1
-0.5
0
0.5
1
Spearman r

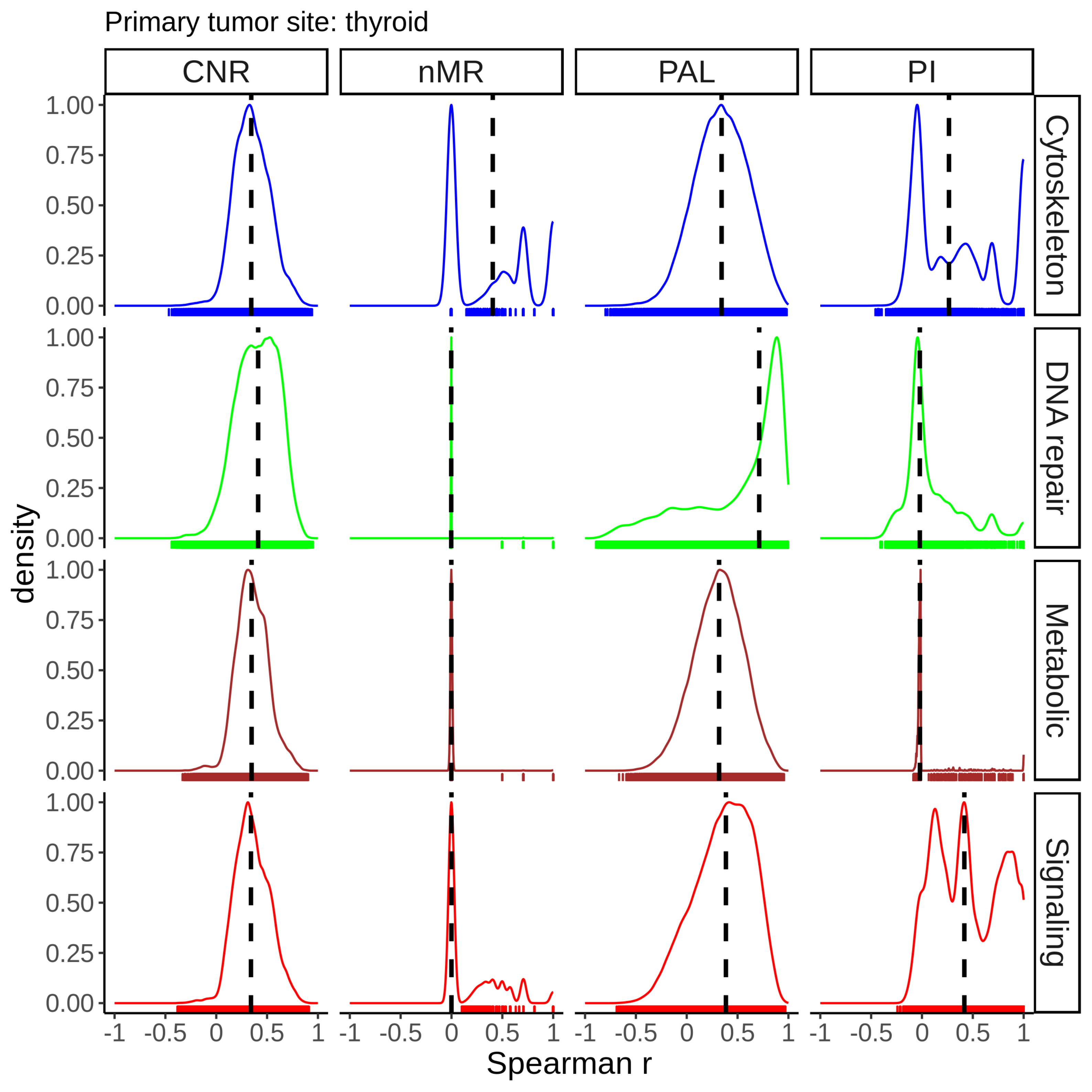

Primary tumor site: thyroid
CNR
nMR
PAL
PI
Cytoskeleton
DNA repair
Metabolic
Signaling
density
1.00
0.75
0.50
0.25
0.00
-1
-0.5
0
0.5
1
Spearman r

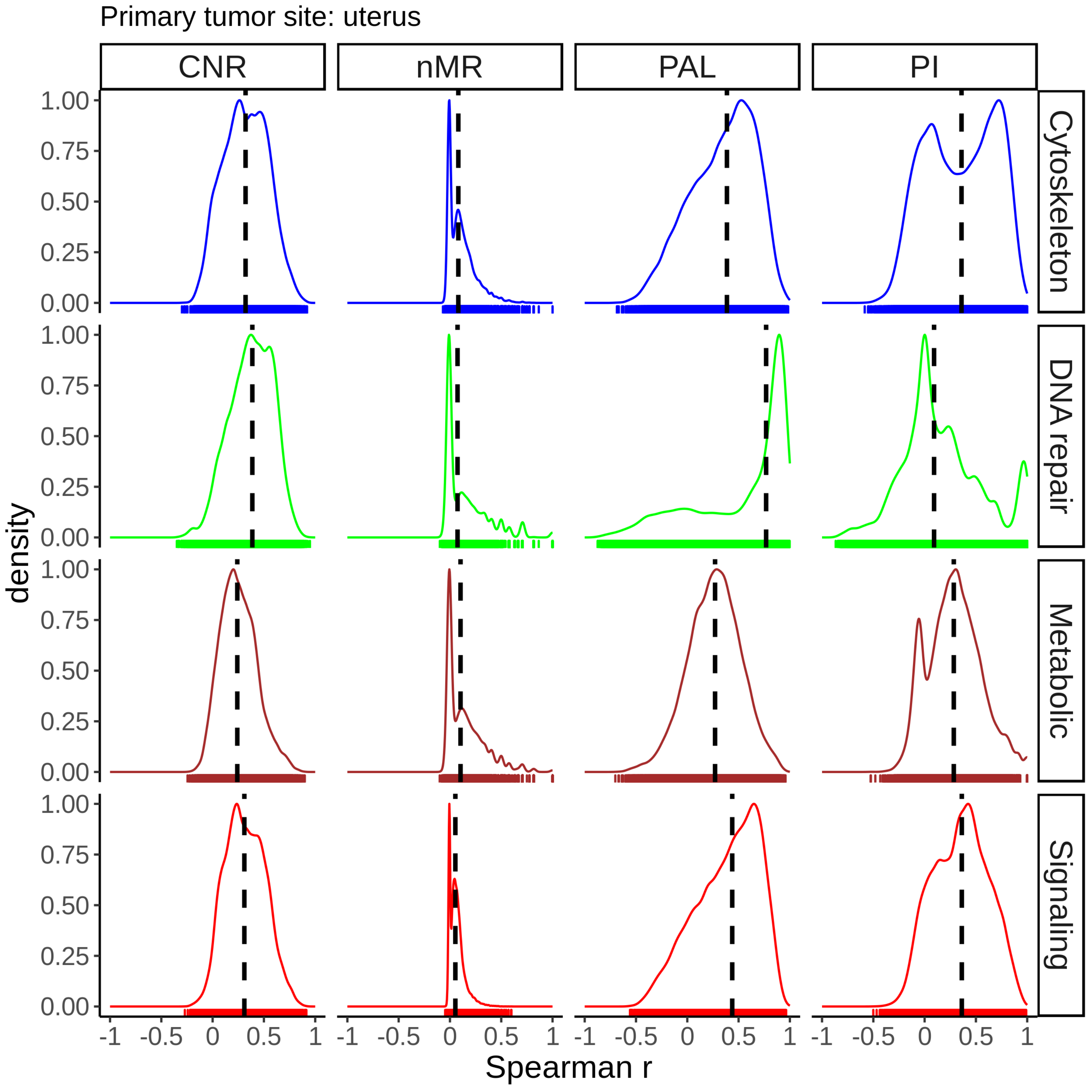

Primary tumor site: uterus
CNR
nMR
PAL
PI
Cytoskeleton
DNA repair
Metabolic
Signaling
density
Spearman r
1.00
0.75
0.50
0.25
0.00
-1
-0.5
0
0.5
1
