# Supplementary material for: Mutation Enrichment and Transcriptomic Activation Signatures of 419 Molecular Pathways in Cancer
Source: Cancers (Basel). 2020 Jan 22;12(2):271. doi: 10.3390/cancers12020271 (PMC7073226; doi:10.3390/cancers12020271)

Average Binary alteration score(BAC) per gene and tumor subtype

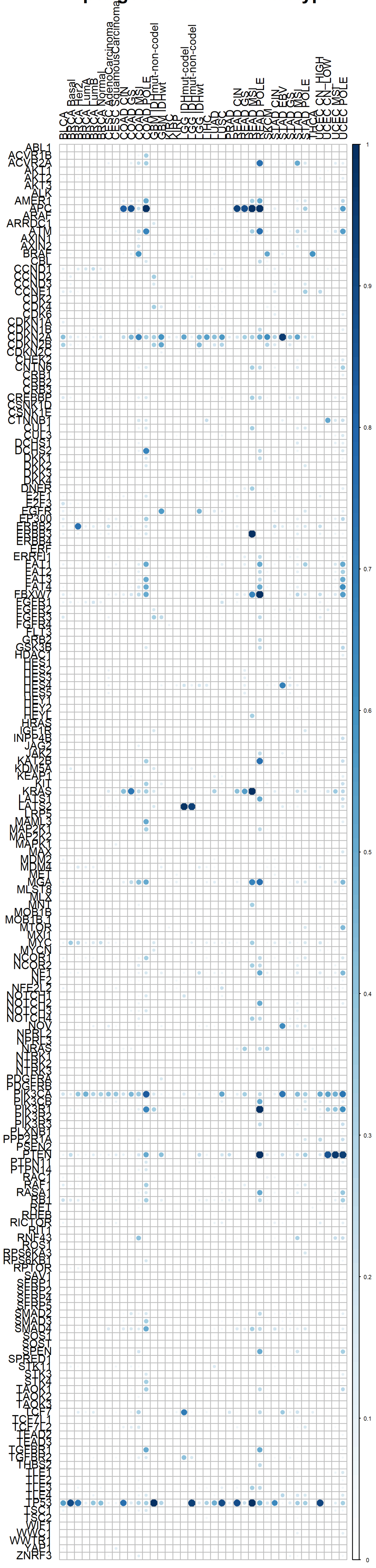

Average logarithmic Case-to-norm ratio(lg(CNR)) per gene and tumor subtype

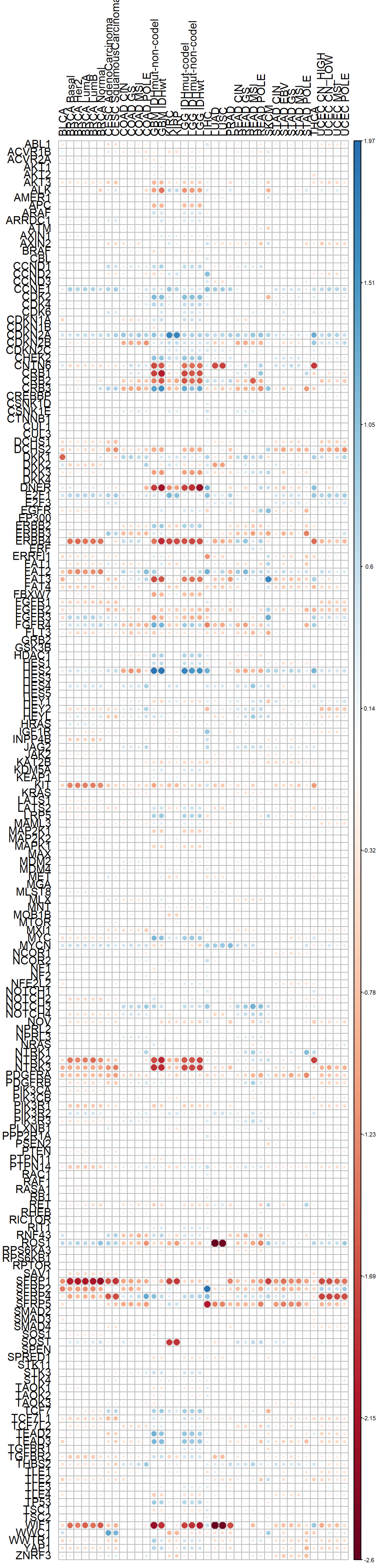

Average logarithmic Normalized mutation rate (lg(nMR)) per gene and tumor subtype

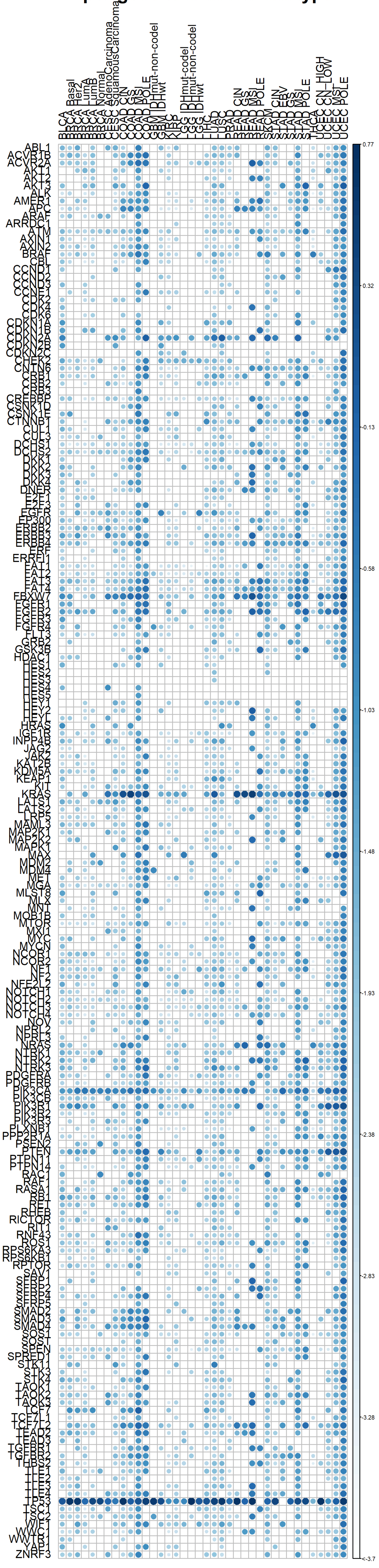

Supplement: Supplementary file 1 [file cancers-12-00271-s001.zip › supplementary revised/Supplementary_File2.pdf]
